# Identifying profiles of brain structure and associations with current and future psychopathology in youth

**DOI:** 10.1016/j.dcn.2021.101013

**Published:** 2021-09-14

**Authors:** Matthew Mattoni, Sylia Wilson, Thomas M. Olino

**Affiliations:** a Temple University, Department of Psychology, 1701 N 13th St, Philadelphia, PA 19122, USA; b University of Minnesota, Institute of Child Development, 1 E River Rd, Minneapolis, MN, 55455, USA

**Keywords:** Brain profile, Brain structure, Latent profile analysis, Youth psychopathology, ABCD study

## Abstract

Brain structure is often studied as a marker of youth psychopathology by examining associations between volume or thickness of individual regions and specific diagnoses. However, these univariate approaches do not address whether the effect of a particular region may depend on the structure of other regions. Here, we identified subgroups of individuals with distinct profiles of brain structure and examined how these profiles were associated with concurrent and future youth psychopathology. We used latent profile analysis to identify distinct neuroanatomical profiles of subcortical region volume and orbitofrontal cortical thickness in the ABCD study (*N* = 9376, mean age = 9.91, *SD* = 0.62). We identified a five-profile solution consisting of a reduced subcortical volume profile, a reduced orbitofrontal thickness profile, a reduced limbic and elevated striatal volume profile, an elevated orbitofrontal thickness and reduced striatal volume profile, and an elevated orbitofrontal thickness and subcortical volume profile. While controlling for age, sex, and intracranial volume, profiles exhibited differences in concurrent psychopathology measured dimensionally and categorically and in psychopathology at 1-year follow-up measured dimensionally. Results show that profiles of brain structure have incremental validity for associations with youth psychopathology beyond intracranial volume.

## Highlights

• Latent Profile Analysis identified distinct profiles of brain structure in youth.
• Profiles consider multiple brain regions jointly, rather than each independently.
• Profiles differed in multiple current and future forms of psychopathology.
• Differences in psychopathology were beyond age, sex, and intracranial volume.

## 1 Introduction

Numerous studies have identified associations between cortical and subcortical brain structure and psychopathology (e.g., depression, Schmaal et al. (2017); anxiety, Schienle et al. (2011); Attention-Deficit/Hyperactivity Disorder (ADHD), Valera et al. (2007); and behavioral disorders, Fairchild et al. (2011)). Many studies have relied on adult samples and cross-sectional designs, with fewer studies examining youth with longitudinal designs (e.g., Busso et al., 2017; Zipursky et al., 2011). Furthermore, previous work on anatomical correlates of psychopathology has relied on differences in specific a priori regions and/or massive univariate analyses. However, it may be that the effect of one region on psychopathology is dependent on the structural characteristics of one or multiple other regions, which would require alternative approaches to univariate tests. Here, we examined how structural brain profiles, or patterns of brain structure across multiple regions, may be associated with concurrent or later experience of psychopathology.

Attention to the association between brain structure and psychopathology has varied by type of disorder and use of adult or child samples. Mood and internalizing disorders have been commonly studied in adults, with inconsistent findings reported for the presence or direction of effects. Meta-analyses and review of depression in adults have found decreased volume in subcortical regions including the hippocampus, putamen, caudate, amygdala, pallidum, insula, and thalamus (Koolschijn et al., 2009, Lorenzetti et al., 2009, Peng et al., 2016) and cortical thickness abnormalities in orbitofrontal, ventromedial, and anterior cingulate regions (Li et al., 2020, Lorenzetti et al., 2009). Although there are fewer studies of youth, similar associations have been identified in smaller youth samples (Caetano et al., 2007, Merz et al., 2018, Rosso et al., 2005) and in a large sample of both adults and children (Schmaal et al., 2016, Schmaal et al., 2017). Bipolar disorder in youth has similarly been associated with decreased volume in the hippocampus, amygdala, and cortical regions, but increased volume in the nucleus accumbens (Ahn et al., 2007, Blumberg et al., 2003, Frazier et al., 2005).

There are comparatively fewer large studies or meta-analyses of neuroanatomical correlates of anxiety and results have been mixed. In adults, studies have found associations between anxiety disorders and greater volume in the striatum, amygdala, and dorsomedial prefrontal cortex, and decreased volume in the hippocampus, insula, and thalamus, and thickness differences in various cortical regions (Bas-Hoogendam et al., 2017, Frick et al., 2013, Hilbert et al., 2015, Moon et al., 2014, Schienle et al., 2011, Syal et al., 2012). In youth, anxiety disorders have been associated with increased prefrontal cortical thickness, increased putamen volume, and decreased hippocampus volume (Gold et al., 2017, Liao et al., 2013).

Studies of youth brain structure and externalizing disorders have also found associations with both cortical and subcortical regions. ADHD has been well studied in youth, as a meta-analysis of youth studies and a large sample of youth and adults reported associations between ADHD and smaller volumes across cortical regions and the accumbens, amygdala, caudate, hippocampus, and putamen (Hoogman et al., 2017, Valera et al., 2007). There are fewer studies of disruptive behavioral disorders, such as conduct disorder. In a recent large study, Waller et al. (2020) found associations between disruptive and callous traits and decreased volume in the amygdala, insula, hippocampus, superior frontal gyrus, and anterior cingulate cortex. Fairchild et al. (2011) reported similar findings in a study with a small sample of youth with conduct disorder. For prodromal psychosis, Brent et al. (2013) reviewed studies of brain structure in youth with clinically high-risk symptoms for psychosis, reporting associations with cortical thinning in the frontal lobe and volumetric differences in the amygdala, hippocampus, and thalamus.

Overall, various forms of youth psychopathological disorders have been associated with brain structure, typically by decreased volumes, in multiple regions. These studies provide foundational support for associations between cortical and subcortical structure and psychopathology. However, the presence and direction of regional effects are inconsistent between studies (Koolschijn et al., 2009, McDonald et al., 2004, Valera et al., 2007). This may be partially explained by the reliance on confirmatory region of interest (ROI) and/or whole-brain massive univariate approaches that test each brain region independently. While this approach considers each region’s structure as an individual risk factor, the brain is highly dynamic such that individual regions do not have absolute function but are functionally interdependent with other regions (Pessoa, 2014). Therefore, it is likely that risk conferred by the structure of one region is dependent on the structure of others. In univariate analyses, there may be overall group differences in multiple regions. However, independent sets of participants within each group may be driving differences across individual brain regions.

Instead, patterns of brain structure across multiple interdependent regions, akin to a multidimensional interaction of several subcortical and cortical regions, may demonstrate greater specificity of associations with psychopathology. Some studies have taken this approach to find that interactions between the structure of multiple regions are associated with psychopathology. Ameis et al. (2014) found that while amygdala volume was not independently associated with externalizing behaviors, an interaction of amygdala-orbitofrontal cortex structure was associated with externalizing problems. Furthermore, longitudinal work with adolescents has found that amygdala-cortical structural coupling is associated with increases in aggression over time (Roberts et al., 2021), as well as differences in depressive symptoms (Vijayakumar et al., 2017) and externalizing behaviors (Bos et al., 2018), indicating that the effect of cortical structure on psychopathology is moderated by amygdala structure.

While these studies found interactions between the structure of two regions, interpretations for this approach would become difficult with the inclusion of multiple additional regions. Rather than modeling interactions, latent profile analysis (LPA) can identify discrete profiles of brain structure, enabling simultaneous modeling of numerous regions. In this alternative approach, subgroups of individuals with common structural patterns can be identified as neuroanatomic profiles to be compared on clinical phenotypes, testing the effect of multiple regions modeled jointly, rather than independently. For example, rather than testing the effect of caudate volume, differences in a structural pattern across multiple striatal and prefrontal regions can be associated with psychopathology. Previous work in the Adolescent Brain Cognitive Development (ABCD) study used latent profile analysis for this multidimensional approach with functional, but not structural, imaging and identified neurodevelopmental subgroups characterized by distinct reward, inhibition, and emotion regulation patterns that displayed differences in clinical outcomes (Lichenstein et al., 2021). However, parallel work has not yet been done using brain structure.

Here, we used data from the ABCD study (Casey et al., 2018) to identify profiles of youth with similar structural patterns and examine how distinct profiles may be associated with psychopathology. The ABCD study is a large, multisite, population-based sample that is well characterized on brain structure and concurrent and future psychopathology (Barch et al., 2018, Garavan et al., 2018, Hagler et al., 2019). We used latent profile analysis to identify subgroups of individuals based on patterns of brain structure for both cortical and subcortical regions frequently implicated in psychopathology. While we focused our selection on regions often implicated in youth psychopathology, we also included regions with common associations in adult psychopathology due to a lack of large studies or meta-analyses for many disorders in youth (Table 1). Specifically, profiles were derived using 18 total neuroanatomical indicators, including volume measures for the bilateral accumbens, amygdala, caudate, hippocampus, pallidum, putamen, thalamus and thickness measures for 2 bilateral orbitofrontal cortex (OFC) regions. The OFC was included due to its frequent associations with youth psychopathology and interactions with subcortical regions in psychological functions such as reward processing and emotion regulation (Kringelbach, 2005). After deriving latent profiles, we compared profiles on cross-sectional youth diagnoses and dimensions of psychopathology and one-year follow-up dimensional assessments of psychopathology. We compared profiles to dimensional clinical symptoms in addition to categorical diagnoses as dimensional approaches may better assess the nature of psychopathology as they can identify gradations of subclinical symptoms (Markon et al., 2011). In a previous report from the ABCD study, a general psychopathology factor was associated with globally smaller gray matter volumes, considered independently, though most of these associations were lost after controlling for intracranial volume (ICV; Durham et al., 2021). Here, we test if profiles of brain structure are associated with youth psychopathology, beyond ICV.

**Table 1:** Regional associations with various forms of psychopathology.

Region	Bipolar Disorder	Depression	Anxiety	ADHD	Behavioral Disorders	Prodromal Psychosis
Accumbens	Ahn et al., 2007^a^		Hilbert et al., 2015	Hoogman et al., 2017^a^		
Amygdala	Blumberg et al., 2003^a^	Lorenzetti et al., 2009;Rosso et al., 2005^a^;Schmaal et al., 2016^a^	Schienle et al., 2011	Hoogman et al., 2017^a^	Fairchild et al., 2011^a^;Waller et al., 2020^a^	Brent et al., 2013^a^
Caudate		Koolschijn et al., 2009;Lorenzetti et al., 2009	Hilbert et al., 2015	Hoogman et al., 2017^a^;Valera et al., 2007^a^	Fairchild et al., 2011^a^	
Hippocampus	Blumberg et al., 2003^a^;Frazier et al., 2005^a^	Caetano et al., 2007^a^;Koolschijn et al., 2009;Li et al., 2020;Lorenzetti et al., 2009;Merz et al., 2018^a^;Schmaal et al., 2016^a^	Gold et al., 2017^a^;Moon et al., 2014	Hoogman et al., 2017^a^	Waller et al., 2020^a^	Brent et al., 2013^a^
Orbitofrontal Cortex		Koolschijn et al., 2009;Lorenzetti et al., 2009;Merz et al., 2018^a^; Schmaal et al., 2017^a^	Frick et al., 2013;Gold et al., 2017^a^;Syal et al., 2012;	Valera et al., 2007^a^	Fairchild et al., 2011^a^;Waller et al., 2020^a^	Brent et al., 2013^a^
Putamen		Koolschijn et al., 2009	Bas-Hoogendam et al., 2017;Hilbert et al., 2015;Liao et al., 2013^a^	Hoogman et al., 2017^a^		
Pallidum		Lorenzetti et al., 2009;Merz et al., 2018^a^		Valera et al., 2007^a^		
Thalamus		Peng et al., 2016	Moon et al., 2014			Brent et al., 2013^a^

a Includes sample of children and/or adolescents

## 2 Methods

The study preregistration is available on Open Science Framework (www.osf.io/d57qu). Deviations from the preregistration are that we examined OFC thickness rather than OFC volume, following previous recommendations (Schwarz et al., 2016, Winkler et al., 2010), we residualized volumes using ICV and controlled for ICV rather than total brain volume for consistency with previous ABCD studies, and we added assessment of PDQ and follow-up scores as secondary analyses. Additionally, we ran supplemental analyses using the originally preregistered variables, which are presented in the Supplementary Materials.

### 2.1 Participants

The sample consisted of 11,875 9- to 10-year-old youth (47.8% female, *M*_*age*_ = 9.91) and their parents measured at baseline in the ABCD Study (Release 3.0), a 21-site study examining how neurobiological and environmental changes may influence youth health and functioning over time. Full details on recruitment and sample characteristics are available elsewhere (Garavan et al., 2018). The demographically diverse, population-based sample was recruited to mirror demographic norms from the American Community Survey (ACS) with regard to race, ethnicity, sex, SES, and urban/rural residency. Recruitment was primarily school based, with approximately 10% of the sample recruited through additional means (e.g., mailing lists, referrals from already enrolled study participants, summer activity outreach, and twin identification from birth registries). For this study, participants that did not pass ABCD structural quality control were excluded (Hagler et al., 2019). One participant per family was then randomly selected for inclusion. The final sample in this study included *N* = 9376 youth (*M*_age_ = 9.91, 47.5% female at birth). Sample selection is detailed in Supplementary Fig. 1.

### 2.2 Measures

#### 2.2.1 Imaging acquisition and processing

Full details regarding imaging acquisition, preprocessing, and quality control are available elsewhere (Casey et al., 2018, Hagler et al., 2019). Imaging acquisition methods were developed by the ABCD Data Analysis and Informatics Center (DAIC) and the ABCD Imaging Acquisition Workgroup. Imaging methods and assessments were selected, optimized, and harmonized across all 21 sites to measure brain structure and function relevant to adolescent development and addiction. The 21 sites used 3T scanner platforms (*Siemens Prisma, General Electric (GE) 750 and Philips)* and multi-channel coils capable of multiband echo planar imaging (EPI) acquisitions, using a standard adult-size coil.

The ABCD DAIC performed centralized processing and analysis of MRI data within the Multi-Modal Processing Stream (MMPS), a software package developed and maintained in-house at the Center for Multimodal Imaging and Genetics (CMIG) at the University of California, San Diego (UCSD). Processing steps included correction for distortion and head motion, cortical surface reconstruction and subcortical segmentation using FreeSurfer v5.3, automated, atlas-based subcortical structure labeling, derivation of morphometric measures (including cortical thickness), and post-processing QC (manual review by trained technicians for motion, intensity inhomogeneity, white matter underestimation, pial overestimation, and magnetic susceptibility artifact). We included participants that met all manual and automated postprocessing quality control criteria (799 participants, 6.7% of sample, failed QC). We extracted data from 18 total regions of interest (ROIs), including volume measures for 7 bilateral subcortical regions (accumbens area, amygdala, caudate, hippocampus, putamen, pallidum, thalamus-proper) and thickness measures for 2 bilateral cortical regions (lateral OFC, medial OFC). We also extracted ICV as a covariate for model creation and profile comparisons.

#### 2.2.2 Measures of psychopathology

Full details on mental health outcomes in the ABCD study are available elsewhere (Barch et al., 2018, Karcher and Barch, 2021). Dimensional assessment of youth psychopathology was measured using the computerized parent-reported Childhood Behavioral Checklist (CBCL) and the child-reported Prodromal Questionnaire-Brief Version (PDQ). The CBCL and PDQ are both administered annually in the ABCD study (Barch et al., 2018). We examined CBCL and PDQ measures at baseline and one-year follow up, as these waves were fully assessed in the ABCD 3.0 release. The CBCL (Achenbach, 2009) contains 119 items on problem behaviors in childhood scored as 0 = Not True, 1 = Somewhat or Sometimes True, and 2 = Very True or Often True. We compared profiles on mean scores on five revised clinical dimensions previously identified in the ABCD CBCL sample through factor analysis (Michelini et al., 2019). The internalizing factor contained 23 items reflecting anxiety and depression symptoms (α = 0.91 (Baseline),.90 (Follow up)). The externalizing factor contained 55 items reflecting aggressive, oppositional, and conduct issues (α = 0.96,.96). The detachment factor contained 5 items reflecting social withdrawal (α = 0.84,.83). The somatoform factor contained 13 items reflecting bodily concerns and complaints (α = 0.84,.85). The neurodevelopmental factor contained 31 items reflecting inattention, hyperactivity, daydreaming, and clumsiness (α = 0.93,.93). The PDQ measures youth psychosis-proneness by assessing the number of prodromal symptoms (α = 0.95,.96) and symptom severity (α = 0.95,.96) (Karcher et al., 2018, Loewy et al., 2011).

Lifetime diagnoses of youth psychopathology were assessed using the computerized parent-report KSADS. We focused on broad diagnostic categories of depressive disorders (Major Depressive Disorder, Dysthymia, and/or Depression NOS), bipolar disorders (Bipolar I, Bipolar II, and/or Bipolar NOS), anxiety disorders (Generalized Anxiety Disorder, Social Anxiety Disorder, Separation Anxiety Disorder, Specific Phobia, and/or Anxiety NOS), obsessive-compulsive disorder (OCD; and/or OCD NOS), attention deficit hyperactivity disorder (ADHD; and/or ADHD NOS), and behavioral disorders (Oppositional Defiant Disorder and/or Conduct disorder). For each category, youth were scored as 0 = Absent lifetime or 1 = Present lifetime based on meeting criteria for any past or present KSADS diagnosis. The KSADS is administered every two years and full data is only available for the baseline wave in the ABCD 3.0 release, so we did not examine follow up diagnostic assessments.

### 2.3 Statistical analysis

#### 2.3.1 Profile estimation

Models were estimated using robust maximum likelihood estimation and accounted for the multisite design using TYPE = COMPLEX option and weighted according to probability sampling weights. LPA models were estimated on 18 indicators of subcortical volume residuals and OFC thickness measures using Mplus 8.4 (Muthén and Muthén, 1998). Subcortical volume measures were residualized using ICV and standardized to have a mean of 0 and standard deviation of 1 in the LPA. Cortical thickness values were standardized, but not residualized with ICV, following field recommendations (Barnes et al., 2010). Empirical comparisons of models were based on the Akaike Information Criteria (AIC), corrected AIC (AICC), Bayesian Information Criteria (BIC), sample-size adjusted BIC (aBIC), and entropy. Lower BIC and AIC values indicate better fit. Higher entropy values indicate better precision of profile membership assignment. Simulation work (Nylund et al., 2007) found that the BIC performed best of the information criteria. Thus, this criterion is weighted most strongly in empirical comparisons within model sets. We also examined the Lo-Mendell-Rubin Likelihood Ratio Test (LMR-LRT) that compares whether the *k-*profile solution is a significantly better fit to the data than the *k – 1* profile solution. All models were estimated with a sufficient number of random starts to yield a replicated log-likelihood value. All subcortical ROI volume residuals and OFC ROI thickness measures were standardized using the full sample such that interpretations of profiles can be described as deviations in standard deviation units.

#### 2.3.2 Profile comparisons

Profile comparisons on outcomes were implemented using the manual three-step approach recommended by Asparouhov and Muthén (2014). As with the profile derivation, models were estimated using robust maximum likelihood estimation and accounted for the multisite design using TYPE = COMPLEX option and were weighted according to probability weights. We relied on this approach to compare profiles as we were interested in profile differences on outcomes when including covariates in the model. This approach estimates profile differences on outcomes using a pseudo-profile draw using posterior probabilities. When there was evidence that there was an omnibus difference in outcomes, we examined pairwise comparisons on outcomes across profiles. Profiles were compared on dimensional measures of CBCL factors and PDQ scores at baseline and 1-year follow up and categorical measures of broad lifetime KSADS diagnosis at baseline. Tests of profile differences across three families (baseline CBCL, baseline KSADS, follow-up CBCL) were adjusted using the Benjamini-Hochberg false discovery rate correction to control for multiple comparisons. Statistical analyses were conducted with a priori covariates of age, sex assigned at birth, and ICV. Age and sex were included as a covariate in consistency with previous ABCD structural studies (Durham et al., 2021, Waller et al., 2020) and due to previous findings of sex differences in brain structure and psychopathology (Lorenzetti et al., 2009). For profile comparisons of follow-up CBCL and PDQ scores, we also controlled for the same measure at baseline. Data with missing outcomes or covariates were listwise deleted.

## 3 Results

### 3.1 Latent profiles

We conducted LPAs with 18 structural indicators (7 bilateral subcortical volume residuals and 2 bilateral OFC thickness measures) that specified 2–9 profiles. All solutions were admissible and global minima were achieved. No solution contained a profile with less than 3% of the total sample, so no solutions were rejected based on very low prevalence. Model fit information is presented in Table 1. There were no minima identified for any information criteria and the LMR-LRT did not identify significant differences in model fit between solutions. Thus, there is little statistical information suggesting the preference of a single profile solution. Model selection was theoretically informed by balancing parsimony of model solutions with the observation of distinct profiles of brain region structure.

Across solutions, profiles exhibited near lateral symmetry. Solutions with 2, 3, and 4 profiles were characterized by profiles with similarities in relative sizes within cortical and subcortical groupings, such that within each profile, all OFC ROIs were of similar relative size and all subcortical ROIs were of similar relative size. Beginning with the 5-profile solution, profiles demonstrated qualitative, rather than quantitative, differences in subcortical region sizes across profiles, such that there was variability between relative sizes of different subcortical regions within profiles. We focused on the 5-profile solution as it contained profiles that had distinct qualitative groupings of OFC, striatal, and limbic regions, but is more parsimonious than solutions with 6–9 profiles. We describe the 5-profile solution based on qualifying deviations of at least .30 standard deviations from the grand mean for each indicator. The Supplementary materials provide additional details on alternative solutions and how membership in profiles transitions across solutions (Supplementary Figs. 2–4).

**Fig. 1:**
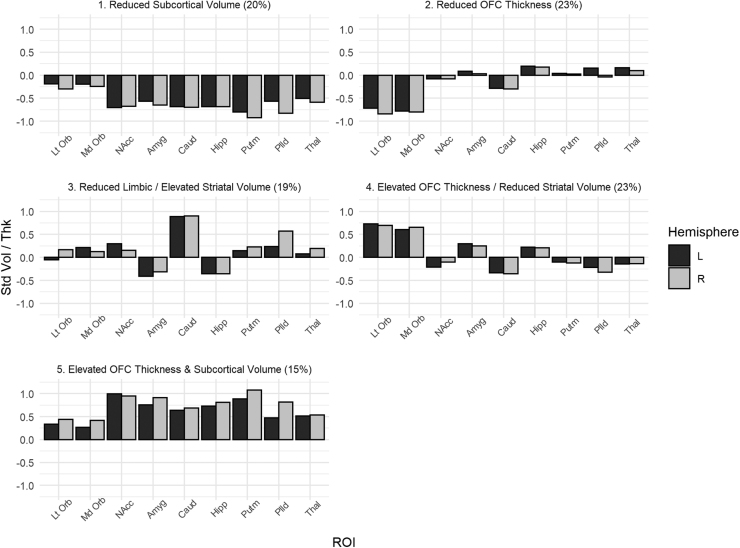
*5-Profile Solution* Note. y-axis represents standardized structural measures for each ROI for each profile. Abbreviations: Std Vol/Thk = Standardized Volume Residual/Thickness; Lt Orb = Lateral Orbitoftronal Cortex, Md Orb = Medial Orbitofrontal Cortex, NAcc = Nucleus Accumbens, Amyg = Amygdala, Caud = Caudate, Hipp = Hippocampus, Putm = Putamen, Plld = Pallidum, Thal = Thalamus.

The 5-profile solution is shown in Fig. 1. Profile 1 included 20% of sample and was characterized by bilaterally reduced subcortical volumes (− 0.56 to − 0.92). We refer to this as the reduced subcortical volume profile. Profile 2 included 23% of the sample and was characterized by reduced bilateral OFC thicknesses (− 0.72 to − 0.84). We refer to this as the reduced OFC thickness profile. Profile 3 included 19% of the sample and was characterized by bilaterally reduced amygdala and hippocampus volumes (− 0.31 to − 0.41) but elevated bilateral caudate volumes (Left,.89; Right,.90) and right pallidum volume (0.57). We refer to this as the reduced limbic/elevated striatal volume profile. Profile 4 included 23% of the sample and was characterized by bilaterally elevated OFC thicknesses (0.61–0.73), bilaterally reduced caudate volume (Left, − 0.33; Right, − 0.36), and reduced right pallidum volume (− 0.32). We refer to this as the elevated OFC thickness/reduced striatal volume profile. Profile 5 included 15% of the sample was characterized by bilaterally elevated subcortical volumes (0.47–1.08) and bilaterally elevated OFC thicknesses (0.34–0.44), though the left medial OFC did not reach threshold (0.27). We refer to this as the elevated OFC thickness and subcortical volume profile.

### 3.2 Profile comparisons

Profiles significantly differed by sex, age, and ICV. Distributions of these characteristics by profile are displayed in Table 2. The elevated OFC thickness and subcortical volume profile was 36% female; other profiles were nearly 50% female (47.8–55.7%). On average, all profiles represented individuals near 10-years old (9.84–10.05). The elevated OFC thickness/reduced striatal volume profile had the relatively smallest ICV (1486 cm^3^) and the reduced OFC thickness profile had the relatively largest ICV (1522 cm^3^); all profiles represented individuals with an ICV near 1500 cm^3^ (Table 3).

**Table 2:** LPA Fit criteria.

Solution	AIC	BIC	aBIC	AICC	Parameters	Entropy	LMR-LRT *p*
**2**	462954	463347.1	463172.3	462954.7	55	0.774	0.3638
**3**	458446.1	458974.9	458739.7	458447.3	74	0.752	0.6081
**4**	454439.1	455103.7	454808.2	454441.0	93	0.751	0.6034
**5**	**451511.9**	**452312.3**	**451956.3**	**451514.6**	**112**	**0.752**	**0.5079**
**6**	449625.0	450561.1	450144.8	449628.7	131	0.759	0.3380
**7**	448115.3	449187.2	448710.5	448120.2	150	0.755	0.5742
**8**	446752.8	447960.5	447423.5	446759.1	169	0.761	0.5815
**9**	445506.2	446849.7	446252.2	445514.0	188	0.769	0.5289

*Note.* The 5-profile solution was selected based on conceptual differences between profiles and greater parsimony than solutions with more profiles, as there was not a statistically preferred solution. *Abbreviations*: Akaike Information Criteria (AIC), Bayesian Information Criteria (BIC), sample-size adjusted BIC (aBIC), corrected AIC (AICC), Lo-Mendell-Rubin Likelihood Ratio Test (LMR-LRT)

**Table 3:** Profile characteristics.

	1: Reduced Subcortical Volume %/*M* (*SE*)	2: Reduced OFC Thickness %/*M* (*SE*)	3: Reduced Limbic/Elevated Striatal Volume %/*M* (*SE*)	4: Elevated OFC Thickness/Reduced Striatal Volume %/*M* (*SE*)	5: Elevated OFC Thickness & Subcortical volume %/*M* (*SE*)	Wald χ^2^
% Female at Birth	55.7% (0.014)	48.4% (0.015)	47.8% (0.013)	51.1% (0.018)	36.0% (0.022)	66.87***
Age in years	9.98 (0.029)	10.05 (0.029)	9.85 (0.032)	9.84 (0.037)	9.92 (0.032)	48.18**
ICV in cm^3^	1509 (9.74)	1522 (13.72)	1502 (24.37)	1486 (15.81)	1508 (18.67)	18.22**

*Note*. * p < .05. ** p < .01. *** p < .001. ICV = intracranial volume.

Profiles were compared on dimensional and diagnostic clinical assessments. Statistical significance tests of omnibus profile comparisons were adjusted using the Benjamini-Hochberg FDR correction. Profile pairwise comparisons for measures with a significant omnibus test are reported at α = 0.05. Group comparisons also controlled for age, sex, and ICV.

For parent-report of youth dimensions of current psychopathology (Table 4), significant differences among profiles were observed for the internalizing factor, externalizing factor, detachment factor, and neurodevelopmental factor. All omnibus effect sizes are very small (ω^2^s < 0.01). No omnibus significant differences between profiles were found for the somatoform factor, PDQ sum score, or PDQ severity score (all *ps* > 0.05).

**Table 4:** Profile comparisons on dimensional psychopathology.

Measure	1: Reduced Subcortical Volume *M (SD)*	2: Reduced OFC Thickness *M (SD)*	3: Reduced Limbic/Elevated Striatal Volume *M (SD)*	4: Elevated OFC Thickness/Reduced Striatal Volume*M (SD)*	5: Elevated OFC Thickness & Subcortical Volume *M (SD)*	Wald χ^2^
Internalizing	2.87 (3.27)_a_	2.45 (2.89)_b_	2.65 (3.14)_a,b_	2.49 (2.86)_b_	2.39 (2.78)_b_	18.27**
Externalizing	5.40 (5.97)_a_	4.70 (5.86)_b_	4.46 (5.35)_b_	4.58 (5.61)_b_	4.15 (5.44)_b_	13.85*
Detachment	0.87 (1.42)_a_	0.67 (1.09)_b,c_	0.78 (1.36)_a,b_	0.64 (1.11)_c_	0.62 (1.07)_c_	30.73***
Somatoform	1.23 (1.64)	1.12 (1.59)	1.20 (1.62)	1.19 (1.63)	1.08 (1.58)	6.82
Neurodevelop.	3.60 (4.00)_a_	3.21 (3.82)_b_	2.91 (3.65)_b_	2.91 (3.39)_b_	2.50 (3.19)_c_	46.02***
PDQ Sum	2.77 (3.51)	2.54 (3.45)	2.77 (3.42)	2.60 (3.59)	2.78 (3.74)	3.07
PDQ Severity	6.85 (10.97)	6.29 (10.60)	6.58 (9.97)	6.31 (10.80)	6.67 (11.72)	1.82

*Note*. * p < .05. ** p < .01. *** p < .001. For each row with a significant omnibus test, significant post-hoc differences are reflected by differing subscripts. Neurodevelop. = Neurodevelopmental. Degrees of Freedom: df = 4, 9363 for all tests.

For parent-report youth internalizing problems, pairwise comparisons found that the reduced subcortical volume profile had significantly higher scores than the reduced OFC thickness profile (d = 0.07), the elevated OFC thickness/reduced striatal volume profile (d = 0.07), and the elevated OFC thickness and subcortical volume profile (d = 0.07). For externalizing problems, pairwise comparisons found that the reduced subcortical volume profile had significantly high scores than all other profiles (d = 0.05 to.07). For detachment, pairwise comparisons found that the reduced subcortical volume profile had significantly higher scores than the reduced OFC thickness profile (d = 0.06), the elevated OFC thickness/reduced striatal volume profile (d = 0.08), and the elevated OFC thickness and subcortical volume profile (d = 0.09). The reduced limbic/elevated striatal volume profile had significantly higher detachment scores than the elevated OFC thickness/reduced striatal volume profile (d = 0.04) and the elevated OFC thickness and subcortical volume profile (d = 0.05). For neurodevelopmental problems, pairwise comparisons found that the reduced subcortical volume profile had significantly higher scores than all other profiles (d = 0.05 to.13) and the elevated OFC thickness and subcortical volume profile had significantly lower scores than all other profiles (d = 0.04 to.13).

For diagnoses (Table 5), profiles significantly differed on lifetime depression disorder, bipolar disorder, anxiety disorders, and ADHD. All omnibus effect sizes are very small (ω^2^ <0.01). Omnibus differences between profiles were not significant for behavioral disorders or OCD (all *p*s > 0.05).

**Table 5:** Profile comparisons on diagnostic outcomes.

Diagnosis	1: Reduced Subcortical Volume *Prob (CI)*	2: Reduced OFC Thickness *Prob (CI)*	3: Reduced Limbic/Elevated Striatal Volume *Prob (CI)*	4: Elevated OFC Thickness/Reduced Striatal Volume *Prob (CI)*	5: Elevated OFC Thickness & Subcortical Volume *Prob (CI)*	Wald χ^2^
Depression	0.09 (0.08–0.11)_a_	0.06 (0.05–0.08)_b_	0.06 (0.04–0.07)_b_	0.07 (0.06–0.09)_a,b_	0.06 (0.05–0.08)_b_	16.36*
Bipolar	0.10 (0.09–0.12)_a_	0.07 (0.05–0.09)_b_	0.07 (0.06–0.09)_b_	0.07 (0.05–0.09)_b_	0.06 (0.04–0.10)_b_	30.56***
Anxiety	0.42 (0.38–0.46)_a_	0.35 (0.31–0.40)_b,c_	0.40 (0.38–0.43)_a,b_	0.36 (0.32–0.4)_c_	0.38 (0.35–0.41)_b,c_	11.33*
OCD	0.12 (0.10–0.13)	0.12 (0.10–0.15)	0.11 (0.10–0.13)	0.11 (0.09–0.13)	0.12 (0.10–0.15)	2.13
Behavioral	0.17 (0.14–0.19)	0.16 (0.14–0.19)	0.15 (0.12–0.18)	0.15 (0.12–0.19)	0.15 (0.13–0.17)	3.39
ADHD	0.25 (0.22–0.27)_a_	0.23 (0.2–0.27)_a,b_	0.19 (0.17–0.22)_b,c_	0.22 (0.2–0.25)_a,b_	0.17 (0.14–0.2)_c_	38.87***

*Note*. * p < .05. ** p < .01. *** p < .001. For each row, significant post-hoc differences are reflected by differing subscripts. Degrees of Freedom: df = 4, 9363 for all tests.

For depression diagnoses, pairwise comparisons found that the reduced subcortical volume profile had significantly more lifetime diagnoses than the reduced OFC thickness profile (d = 0.06), the reduced limbic/elevated striatal volume profile (d = 0.07), and the elevated OFC thickness and subcortical volume profile (d = 0.05). For bipolar disorder diagnoses, pairwise comparisons found that the reduced subcortical volume profile had significantly more lifetime diagnoses than all other profiles (d = 0.05 to.06). For anxiety diagnoses, pairwise comparisons found that the reduced subcortical volume profile reported significantly more lifetime diagnoses than the reduced OFC thickness profile (d = 0.06), the elevated OFC thickness/reduced striatal volume profile (d = 0.04), and the elevated OFC thickness and subcortical volume profile (d = 0.04). The reduced limbic/elevated striatal volume profile had more lifetime anxiety diagnoses than the elevated OFC thickness/reduced striatal volume profile (d = 0.04). For ADHD diagnoses, pairwise comparisons found that the reduced subcortical volume profile reported significantly more lifetime diagnoses than the reduced limbic/elevated striatal volume profile (d = 0.07) and the elevated OFC thickness and subcortical volume profile (d = 0.11). The elevated OFC thickness and subcortical volume profile also reported significantly fewer lifetime diagnoses than the reduced OFC thickness profile (d = 0.06) and the elevated OFC thickness/reduced striatal volume profile (d = 0.08).

For 1-year follow up scores (Table 6), in addition to age, sex, and ICV, we further controlled for baseline scores of the corresponding factor. We found significant differences between profiles for internalizing, externalizing, somatoform, and neurodevelopmental problems. All omnibus effect sizes are very small (ω^2^ <0.01). No omnibus significant differences between profiles were found for the detachment factor, PDQ sum score, or PDQ severity score (all *p*s > 0.05). Correlation coefficients between baseline and follow-up scores within profiles are presented in Supplementary Table 3.

**Table 6:** Profile comparisons on follow-up dimensional psychopathology.

Measure	1: Reduced Subcortical Volume *M (SD)*	2: Reduced OFC Thickness *M (SD)*	3: Reduced Limbic/Elevated Striatal Volume *M (SD)*	4: Elevated OFC Thickness/Reduced Striatal Volume *M (SD)*	5: Elevated OFC Thickness & Subcortical Volume *M (SD)*	Wald’s χ^2^
Internalizing	2.44 (2.19)_a_	2.58 (2.13)_a,b_	2.54 (2.25)_a,b_	2.59 (2.15)_a,b_	2.73 (2.31)_b_	15.19*
Externalizing	4.26 (3.75)_a,b_	4.50 (3.86)_c_	4.54 (3.61)_b,c_	4.14 (3.58)_a_	4.34 (3.45)_a,b,c_	12.55*
Detachment	0.77 (1.03)	0.74 (0.95)	0.77 (0.98)	0.76 (0.95)	0.72 (0.93)	4.02
Somatoform	1.17 (1.42)_a,b_	1.19 (1.35)_a_	1.15 (1.40)_a,b_	1.01 (1.29)_b_	1.17 (1.33)_a,b_	13.26*
Neurodevelop.	2.85 (2.33)_a,b_	3.06 (2.46)_a,c_	2.97 (2.40)_a,b,c_	2.84 (2.37)_b_	3.11 (2.28)_c_	19.67**
PDQ Sum	2.00 (2.92)	1.95 (2.62)	2.14 (3.01)	1.99 (2.94)	1.85 (2.83)	7.60
PDQ Severity	5.09 (9.19)	4.58 (7.44)	5.13 (9.02)	4.70 (8.61)	4.35 (8.65)	7.89

*Note*. * p < .05. ** p < .01. *** p < .001. Neurodevelop. = Neurodevelopmental. For each row with a significant omnibus test, significant pairwise differences are reflected by values that do not share a common subscript. Follow-up comparisons controlled for baseline scores in the corresponding measure. Degrees of Freedom: df = 4, 8818 for Internalizing, Externalizing, Detachment, and Somatoform tests; df = 4, 8819 for Neurodevelopmental test; df = 4, 8812 for PDQ Sum; df = 4, 8814 for PDQ Severity.

For internalizing problems, follow-up comparisons found that the reduced subcortical volume profile had significantly smaller (See Table 6) scores than the elevated OFC thickness and subcortical volume profile (d = 0.08). For externalizing problems, follow-up comparisons found that the reduced OFC thickness profile had significantly greater scores than the reduced subcortical volume profile (d = 0.04) and the elevated OFC thickness/reduced striatal volume profile (d = 0.04). The reduced limbic/elevated striatal volume profile also had significantly higher externalizing scores than the elevated OFC thickness/reduced striatal volume profile (d = 0.04). For somatoform problems, follow-up comparisons found that the reduced OFC thickness profile reported significantly higher scores than the elevated OFC thickness/reduced striatal volume profile (d = 0.07). For neurodevelopmental problems, follow-up comparisons found that the elevated OFC thickness and subcortical volume profile had significantly higher scores than the reduced subcortical volume profile (d = 0.06) and the elevated OFC thickness/reduced striatal volume profile (d = 0.06). The reduced OFC thickness profile also had greater neurodevelopmental problems than the elevated OFC thickness/reduced striatal volume profile (d = 0.04).

### 3.3 Sensitivity and robustness analysis

Our sample was highly similar in age, sex, and CBCL scores to the larger sample that included siblings (Supplementary Table 4). The LPA using this larger sample was also very similar. There was no preferred solution based on fit criteria (Supplementary Table 5), the 5-Profile solution in this sample was substantively identical to the 5-Profile solution presented here (Supplementary Fig. 5), and each individual’s most likely profile membership was > 95% consistent within each profile between samples (Supplementary Table 6). These results indicate that minimal bias was introduced by including only a single participant per family.

Profile comparisons controlling for additional covariates of MRI model, race/ethnicity, and psychotropic medication status led to substantively similar conclusions, but omnibus tests were no longer significant for profile differences on baseline externalizing (*p* = .433) and follow-up internalizing (*p* = .056) problems.

## 4 Discussion

Previous studies have focused on cortical and subcortical brain structure differences between youth with and without psychopathology, testing each region as an independent marker. In contrast, this study identified profiles of brain structure that were characterized by patterns of multiple indicators spanning subcortical region volumes and orbitofrontal region thicknesses simultaneously. We focused our analysis on the 5-profile solution that showed qualitative differences in the patterns of orbitofrontal thickness and subcortical region brain volumes. The profiles are conceptually plausible, with groupings of orbitofrontal regions, striatal regions, and limbic regions. As our model included relatively few brain regions and did not have a clear statistically preferred solution, we interpret these profiles as structural patterns across clinically relevant regions that may commonly occur in children rather than true subtypes. We found differences between profiles on multiple CBCL dimensions and KSADS lifetime diagnoses, as well as CBCL dimensions at the one-year follow-up after controlling for age, sex, and ICV. Profile differences on dimensional and diagnostic assessments showed similar patterns, increasing confidence in the reliability of results. As previous analysis of regional gray matter volume and dimensional psychopathology in the ABCD study found that most significant relationships were lost after controlling for ICV (Durham et al., 2021), these results may suggest that distinct profiles of brain structural patterns show incremental utility in associations with youth psychopathology compared to regions considered independently.

### 4.1 Implications of brain structure profiles for psychopathology

Here, we discuss interpretations for profile differences in psychopathology. While we refer to differences between individuals in different profiles, we note that individuals are not absolutely assigned to subgroups and profile comparisons accounted for membership probability weighting. The clearest pattern of results showed that youth in the profile characterized by reduced subcortical volume had greater psychopathology across multiple domains, measured both dimensionally and diagnostically, relative to youth in other profiles at baseline. Specifically, they had significantly greater externalizing problems, neurodevelopmental problems, and higher rate of lifetime diagnosis of bipolar disorder than youth in all other profiles. They also had greater internalizing problems, detachment problems, and a higher rate of lifetime anxiety diagnosis than youth in all profiles except those in the reduced limbic/elevated striatal volume profile. Additionally, they had a higher rate of lifetime depression diagnosis than youth in all profiles except the elevated OFC thickness/reduced striatal volume profile and a higher rate of lifetime ADHD diagnosis than youth in the reduced limbic/elevated striatal volume profile and the elevated OFC thickness and subcortical volume profile. These results are consistent with previous ABCD findings of associations between globally smaller gray matter volume and general psychopathology (Durham et al., 2021). Our analysis builds on this study to show that a *pattern* of smaller subcortical gray matter volumes is a particularly robust marker of multiple forms of psychopathology, beyond ICV.

Youth in the elevated OFC thickness and subcortical volume profile had fewer concurrent neurodevelopmental problems than youth in all other profiles, and fewer lifetime ADHD diagnoses than youth in the reduced OFC thickness and the elevated OFC thickness/reduced striatal volume profiles. These convergent results of dimensional and diagnostic measures are consistent with previous findings that youth with ADHD have reduced volumes in subcortical regions, particularly the caudate, and reduced thickness in orbitofrontal regions (Ducharme et al., 2012, Hoogman et al., 2017, Krain and Castellanos, 2006, Narr et al., 2009).

In addition to the consistent patterns of increased concurrent psychopathology for youth in the reduced subcortical volume profile and decreased current neurodevelopmental problems for youth in the elevated OFC thickness and subcortical volume profile, there were multiple nuanced differences between other profiles. The reduced limbic/elevated striatal volume profile had a bilaterally small amygdala and hippocampus, a bilaterally large caudate, and a large right pallidum. In contrast, the elevated OFC thickness/reduced striatal volume profile exhibited the opposite pattern across the caudate, pallidum, amygdala, and hippocampus, though deviations did not reach our threshold for the amygdala (left.29, right.25) or hippocampus (0.22,.21). Comparing these two profiles, youth in the reduced limbic/elevated striatal volume profile had greater concurrent detachment problems and anxiety diagnoses, as well as future externalizing problems at the 1-year follow up. These higher levels of detachment (social withdrawal) problems and anxiety diagnoses may suggest that youth with a pattern of reduced limbic volume *and* elevated striatal profile are at increased risk for social anxiety relative to youth with the opposite pattern of elevated limbic volume and reduced striatal volume. Though findings of structural associations with social anxiety disorder have been mixed (Bas-Hoogendam et al., 2017, Syal et al., 2012, Talati et al., 2013, Wang et al., 2018), a large multi-site study reported increased striatal volume in adults with social anxiety disorder (Bas-Hoogendam et al., 2017). As there have been inconsistent associations between brain structure and social anxiety disorder, further work is needed to examine if structural patterns between striatal, limbic, and orbitofrontal regions are specifically associated with social anxiety. Youth in the reduced limbic/elevated striatal volume profile having increased externalizing problems in 1-year is similar to previous ABCD findings of decreased amygdala volume in youth with conduct disorder cross-sectionally (Waller et al., 2020), though results here may suggest that a pattern across multiple striatal *and* limbic regions has predictive utility.

Finally, youth in the reduced OFC thickness profile had greater externalizing, somatoform, and neurodevelopmental problems at 1-year follow up than youth in the elevated OFC thickness/reduced striatal volume profile. These results may be related to the substantial cortical thinning that occurs during youth brain development (Tamnes et al., 2017), such that youth with decreased orbitofrontal cortex thickness may be at a greater neurodevelopment stage than expected based on age (i.e., a brain-age gap), leaving them at risk for future psychopathology (Cropley et al., 2020). These results suggest that youth with reduced OFC thickness may be at increased risk for future psychopathology in multiple domains.

Profile differences in CBCL scores at 1-year follow-up were largely inconsistent with differences found at baseline. Furthermore, some associations at 1-year follow-up were the opposite direction as baseline associations. Youth in the reduced subcortical volume profile had *fewer* internalizing and neurodevelopmental problems at 1-year follow-up than youth in the elevated OFC thickness and subcortical volume profile and *fewer* externalizing problems than youth in the reduced OFC thickness profile. Similarly reflecting a change in direction, youth in the elevated OFC thickness and subcortical volume profile had *greater* neurodevelopmental problems at 1-year follow-up compared to youth in the reduced subcortical volume and the elevated OFC thickness/reduced striatal volume profiles. These results may suggest that patterns of brain structure at one timepoint may have distinct associations with clinical outcomes at different time points. Alternatively, youth development may be altering brain structure such that associations between structural patterns and psychopathological outcomes change over time. The sample at baseline contains pre-adolescents entering early stages of puberty and there would be changes in brain structure across time. Thus, youth identified in a specific profile at baseline may stay in the same profile, be reclassified into other identified profiles, or may be reclassified into a new profile that is identified at a later time point. Future studies using additional waves of ABCD imaging data can examine how individuals progress from one profile of brain structure to another over time, or how distinct neurodevelopmental patterns are associated with changes in psychopathology.

We did not find profile differences for OCD diagnoses, PDQ sum or severity scores, or behavioral disorder diagnoses. Typical ages of onset for OCD and prodromal symptoms may be later in development than the current sample (Brakoulias et al., 2017; Olsen and Rosenbaum, 2006); thus, associations may manifest at later assessments. Though we did find profile differences in externalizing problems, our lack of significant behavioral diagnostic results conflict with previous ABCD research that associated decreased volume in the amygdala and hippocampus with disruptive behavioral disorders (Waller et al., 2020). One possible explanation is that the previous study assigned participants to a disruptive behavior category by CBCL subscale cutoff score *or* KSADS diagnosis, making it unclear which assessment form drove group differences.

Overall, the identified common patterns explain a significant, but modest proportion of variance in psychopathology beyond global brain volume. Post hoc effect sizes between profiles were small (d = 0.04 to .13) and are similar to those previously reported in case-control studies of youth psychopathology in other samples (e.g. depression, Matsuo et al. (2008) and ADHD, Hoogman et al. (2017)). The effect sizes are also similar to those previously found between individual regions and dimensional psychopathology in the ABCD sample, before controlling for ICV (Durham et al., 2021). However, our results show incremental prediction of profiles beyond the effects of age, sex, and ICV in the same sample.

### 4.2 Limitations and future directions

One strength of this study was preregistration, as making multiple a priori analytic decisions reduces flexibility and the false positive rate (Wicherts et al., 2016). Additionally, the use of a large, diverse, population-based sample and calculation of effect sizes gives confidence in the precision of the detected associations, providing increased ability to generalize findings to the population (Dick et al., 2020). Analytically, the use of LPA considers the constellation of multiple regions simultaneously, which is not feasible with other typical analytical approaches. Furthermore, profile comparison on categorical and dimensional measures of psychopathology were largely consistent, increasing confidence in the reliability of results. Finally, follow-up measures provided information of predictive utility of structural profiles.

However, there are several important limitations that caution our findings. First, statistical indices did not indicate an empirically preferred solution. Though we did not detect an optimal solution within models of 2–9 subgroups, it is possible that a statistically preferred solution could have been detected with more than 9 profiles. However, the interpretability of a very large number of classes would diminish interpretability. As such, identified profiles are interpreted as common structural patterns across clinically relevant regions, rather than true neurodevelopmental subtypes. Full descriptions of alternative solutions are reported in the Supplementary material. Second, ROIs were selected based on previously identified associations with disorders such as depression (Koolschijn et al., 2009), anxiety (Bas-Hoogendam et al., 2017, Schienle et al., 2011), and ADHD (Valera et al., 2007). This ROI selection was driven by both youth and adult literature, as there are fewer large studies or meta-analyses of brain structure and psychopathology in child samples. It is possible that regions associated with youth psychopathology, specifically, were not included in the analysis. Also, reliance on different sets of ROIs may lead to different profiles identified. Thus, others interested in examining alternative sets of ROIs can do so in the present data.

Third, while inclusion of orbitofrontal regions enabled examination of cortical-subcortical developmental mismatch previously identified as a potential risk marker for youth psychopathology (Powers and Casey, 2015), the majority of LPA indicators were subcortical regions. This selection potentially biased models toward differentiating on subcortical features. As such, future work could include more cortical ROIs to define the profiles more comprehensively. Alternative machine learning methods can include many more ROIs flexibly to identify homogenous profiles of brain structure (Varol et al., 2017). Fourth, at the time of this analysis, diagnostic data was not available for the complete cohort at the two-year follow up, so we only included one-year follow up to examine longitudinal prediction. As the ABCD study continues, future work can examine bidirectional associations between profiles of brain structure and an increasing number of domains of psychopathology.

### 4.3 Conclusions

We used latent profile analysis to identify neuroanatomical profiles of subcortical region volume and orbitofrontal cortical thickness in the ABCD study and compared profiles on dimensional and categorical assessments of psychopathology. We identified a five-profile solution with profiles exhibiting distinct patterns across orbitofrontal, striatal, and limbic regions. Overall, results showed differences between structural profiles on concurrent and future assessments of psychopathology, controlling for age, sex, and intracranial volume. Results suggests that distinct structural patterns across subcortical and orbitofrontal brain regions are associated with psychopathology, beyond intracranial volume.

## Declaration of Competing Interest

Mr. Mattoni, Dr. Wilson, and Dr. Olino report no biomedical financial interests or potential conflicts of interest.

## Data Availability

The ABCD data used in this report came from NIMH Data Archive Digital Object Identifier (DOI) 10.15154/1519007. Data are available upon approval from the NIH NDA.

## References

[bib1] Achenbach T. (2009). The Achenbach System of Empirically Based Assessemnt (ASEBA): Development, Findings, Theory, and Applications.

[bib2] Ahn M.S., Breeze J.L., Makris N., Kennedy D.N., Hodge S.M., Herbert M.R., Seidman L.J., Biederman J., Caviness V.S., Frazier J.A. (2007). Anatomic brain magnetic resonance imaging of the basal ganglia in pediatric bipolar disorder. J. Affect. Disord..

[bib3] Ameis S.H., Ducharme S., Albaugh M.D., Hudziak J.J., Botteron K.N., Lepage C., Zhao L., Khundrakpam B., Collins D.L., Lerch J.P., Wheeler A., Schachar R., Evans A.C., Karama S. (2014). Cortical thickness, cortico-amygdalar networks, and externalizing behaviors in healthy children. Biol. Psychiatry.

[bib4] Asparouhov T., Muthén B. (2014). Auxiliary variables in mixture modeling: three-step approaches using Mplus. Struct. Eq. Model. Multidiscip. J..

[bib5] Barch D.M., Albaugh M.D., Avenevoli S., Chang L., Clark D.B., Glantz M.D., Hudziak J.J., Jernigan T.L., Tapert S.F., Yurgelun-Todd D., Alia-Klein N., Potter A.S., Paulus M.P., Prouty D., Zucker R.A., Sher K.J. (2018). Demographic, physical and mental health assessments in the adolescent brain and cognitive development study: rationale and description. Dev. Cogn. Neurosci..

[bib6] Barnes J., Ridgway G.R., Bartlett J., Henley S.M.D., Lehmann M., Hobbs N., Clarkson M.J., MacManus D.G., Ourselin S., Fox N.C. (2010). Head size, age and gender adjustment in MRI studies: a necessary nuisance?. NeuroImage.

[bib7] Bas-Hoogendam J.M., van Steenbergen H., Nienke Pannekoek J., Fouche J.-P., Lochner C., Hattingh C.J., Cremers H.R., Furmark T., Månsson K.N.T., Frick A., Engman J., Boraxbekk C.-J., Carlbring P., Andersson G., Fredrikson M., Straube T., Peterburs J., Klumpp H., Phan K.L., van der Wee N.J.A. (2017). Voxel-based morphometry multi-center mega-analysis of brain structure in social anxiety disorder. NeuroImage Clin..

[bib8] Blumberg H.P., Kaufman J., Martin A., Whiteman R., Zhang J.H., Gore J.C., Charney D.S., Krystal J.H., Peterson B.S. (2003). Amygdala and hippocampal volumes in adolescents and adults with bipolar disorder. Arch. Gen. Psychiatry.

[bib9] Bos M.G.N., Wierenga L.M., Blankenstein N.E., Schreuders E., Tamnes C.K., Crone E.A. (2018). Longitudinal structural brain development and externalizing behavior in adolescence. J. Child Psychol. Psychiatry. Allied Discip..

[bib10] Brakoulias V., Starcevic V., Belloch A., Brown C., Ferrao Y.A., Fontenelle L.F., Lochner C., Marazziti D., Matsunaga H., Miguel E.C., Reddy Y.C.J., do Rosario M.C., Shavitt R.G., Shyam Sundar A., Stein D.J., Torres A.R., Viswasam K. (2017). Comorbidity, age of onset and suicidality in obsessive–compulsive disorder (OCD): an international collaboration. Compr. Psychiatry.

[bib11] Brent B.K., Thermenos H.W., Keshavan M.S., Seidman L.J. (2013). Gray matter alterations in Schizophrenia high-risk youth and early-onset Schizophrenia: a review of structural MRI findings. Child Adolesc. Psychiatr. Clin. N. Am..

[bib12] Busso D.S., McLaughlin K.A., Brueck S., Peverill M., Gold A.L., Sheridan M.A. (2017). Child abuse, neural structure, and adolescent psychopathology: a longitudinal study. J. Am. Acad. Child Adoles. Psychiatry.

[bib13] Caetano S.C., Fonseca M., Hatch J.P., Olvera R.L., Nicoletti M., Hunter K., Lafer B., Pliszka S.R., Soares J.C. (2007). Medial temporal lobe abnormalities in pediatric unipolar depression. Neurosci. Lett..

[bib14] Casey B.J., Cannonier T., Conley M.I., Cohen A.O., Barch D.M., Heitzeg M.M., Soules M.E., Teslovich T., Dellarco D.V., Garavan H., Orr C.A., Wager T.D., Banich M.T., Speer N.K., Sutherland M.T., Riedel M.C., Dick A.S., Bjork J.M., Thomas K.M., ABCD Imaging Acquisition Workgroup (2018). The Adolescent Brain Cognitive Development (ABCD) study: imaging acquisition across 21 sites. Dev. Cogn. Neurosci..

[bib15] Cropley V.L., Tian Y., Fernando K., Mansour L.S., Pantelis C., Cocchi L., Zalesky A. (2020). Brain-predicted age associates with psychopathology dimensions in youths. Biol. Psychiatry Cogn. Neurosci. Neuroimaging.

[bib16] Dick, A.S., Watts, A.L., Heeringa, S., Lopez, D.A., Bartsch, H., Fan, C.C., Palmer, C., Reuter, C., Marshall, A., Haist, F., Hawes, S., Nichols, T.E., Barch, D.M., Jernigan, T.L., Garavan, H., Grant, S., Pariyadath, V., Hoffman, E., Neale, M., Paulus, M.P., Sher, K.J., Thompson, W.K., 2020. Meaningful effects in the adolescent brain cognitive development study, BioRxiv, 2020.09.01.276451. 〈 10.1101/2020.09.01.276451〉.

[bib17] Ducharme S., Hudziak J.J., Botteron K.N., Albaugh M.D., Nguyen T.-V., Karama S., Evans A.C. (2012). Decreased regional cortical thickness and thinning rate are associated with inattention symptoms in healthy children. J. Am. Acad. Child Adoles. Psychiatry.

[bib18] Durham E.L., Jeong H.J., Moore T.M., Dupont R.M., Cardenas-Iniguez C., Cui Z., Stone F.E., Berman M.G., Lahey B.B., Kaczkurkin A.N. (2021). Association of gray matter volumes with general and specific dimensions of psychopathology in children. Neuropsychopharmacology.

[bib19] Fairchild G., Passamonti L., Hurford G., Hagan C.C., von dem Hagen E.A.H., van Goozen S.H.M., Goodyer I.M., Calder A.J. (2011). Brain structure abnormalities in early-onset and adolescent-onset conduct disorder. Am. J. Psychiatry.

[bib20] Frazier J.A., Breeze J.L., Makris N., Giuliano A.S., Herbert M.R., Seidman L., Biederman J., Hodge S.M., Dieterich M.E., Gerstein E.D., Kennedy D.N., Rauch S.L., Cohen B.M., Caviness V.S. (2005). Cortical gray matter differences identified by structural magnetic resonance imaging in pediatric bipolar disorder. Bipolar Disord..

[bib21] Frick A., Howner K., Fischer H., Eskildsen S.F., Kristiansson M., Furmark T. (2013). Cortical thickness alterations in social anxiety disorder. Neurosci. Lett..

[bib22] Garavan H., Bartsch H., Conway K., Decastro A., Goldstein R.Z., Heeringa S., Jernigan T., Potter A., Thompson W., Zahs D. (2018). Recruiting the ABCD sample: design considerations and procedures. Dev. Cogn. Neurosci..

[bib23] Gold A.L., Steuber E.R., White L.K., Pacheco J., Sachs J.F., Pagliaccio D., Berman E., Leibenluft E., Pine D.S. (2017). Cortical thickness and subcortical gray matter volume in pediatric anxiety disorders. Neuropsychopharmacology.

[bib24] Hagler D.J., Hatton S., Cornejo M.D., Makowski C., Fair D.A., Dick A.S., Sutherland M.T., Casey B.J., Barch D.M., Harms M.P., Watts R., Bjork J.M., Garavan H.P., Hilmer L., Pung C.J., Sicat C.S., Kuperman J., Bartsch H., Xue F., Dale A.M. (2019). Image processing and analysis methods for the Adolescent Brain Cognitive Development Study. NeuroImage.

[bib25] Hilbert K., Pine D.S., Muehlhan M., Lueken U., Steudte-Schmiedgen S., Beesdo-Baum K. (2015). Gray and white matter volume abnormalities in generalized anxiety disorder by categorical and dimensional characterization. Psychiatry Res..

[bib26] Hoogman M., Bralten J., Hibar D.P., Mennes M., Zwiers M.P., Schweren L., van Hulzen K.J.E., Medland S.E., Shumskaya E., Jahanshad N., de Zeeuw P., Szekely E., Sudre G., Wolfers T., Onnink A.M.H., Dammers J.T., Mostert J.C., Vives-Gilabert Y., Kohls G., Franke B. (2017). Subcortical brain volume differences of participants with ADHD across the lifespan: an ENIGMA collaboration. Lancet Psychiatry.

[bib27] Karcher N.R., Barch D.M. (2021). The ABCD study: understanding the development of risk for mental and physical health outcomes. Neuropsychopharmacol. Off. Publ. Am. Coll. Neuropsychopharmacol..

[bib28] Karcher N.R., Barch D.M., Avenevoli S., Savill M., Huber R.S., Simon T.J., Leckliter I.N., Sher K.J., Loewy R.L. (2018). Assessment of the prodromal questionnaire–brief child version for measurement of self-reported psychoticlike experiences in childhood. JAMA Psychiatry.

[bib29] Koolschijn P.C.M.P., Haren N.E.M., van, Lensvelt‐Mulders G.J.L.M., Pol H.E.H., Kahn R.S. (2009). Brain volume abnormalities in major depressive disorder: a meta-analysis of magnetic resonance imaging studies. Hum. Brain Mapp..

[bib30] Krain A.L., Castellanos F.X. (2006). Brain development and ADHD. Clin. Psychol. Rev..

[bib31] Kringelbach M.L. (2005). The human orbitofrontal cortex: linking reward to hedonic experience. Nat. Rev. Neurosci..

[bib32] Li Q., Zhao Y., Chen Z., Long J., Dai J., Huang X., Lui S., Radua J., Vieta E., Kemp G.J., Sweeney J.A., Li F., Gong Q. (2020). Meta-analysis of cortical thickness abnormalities in medication-free patients with major depressive disorder. Neuropsychopharmacology.

[bib33] Liao M., Yang F., Zhang Y., He Z., Song M., Jiang T., Li Z., Lu S., Wu W., Su L., Li L. (2013). Childhood maltreatment is associated with larger left thalamic gray matter volume in adolescents with generalized anxiety disorder. PLoS One.

[bib34] Lichenstein S.D., Roos C., Kohler R., Kiluk B., Carroll K.M., Worhunsky P.D., Witkiewitz K., Yip S.W. (2021). Identification and validation of distinct latent neurodevelopmental profiles in the Adolescent Brain and Cognitive Development study. Biol. Psychiatry Cogn. Neurosci. Neuroimaging.

[bib35] Loewy R.L., Pearson R., Vinogradov S., Bearden C.E., Cannon T.D. (2011). Psychosis risk screening with the Prodromal Questionnaire—brief version (PQ-B). Schizophr. Res..

[bib36] Lorenzetti V., Allen N.B., Fornito A., Yücel M. (2009). Structural brain abnormalities in major depressive disorder: a selective review of recent MRI studies. J. Affect. Disord..

[bib37] Markon K.E., Chmielewski M., Miller C.J. (2011). The reliability and validity of discrete and continuous measures of psychopathology: a quantitative review. Psychol. Bull..

[bib38] Matsuo K., Rosenberg D.R., Easter P.C., MacMaster F.P., Chen H.-H., Nicoletti M., Caetano S.C., Hatch J.P., Soares J.C. (2008). Striatal volume abnormalities in treatment-naïve patients diagnosed with pediatric major depressive disorder. J. Child Adoles. Psychopharmacol..

[bib39] McDonald C., Zanelli J., Rabe-Hesketh S., Ellison-Wright I., Sham P., Kalidindi S., Murray R.M., Kennedy N. (2004). Meta-analysis of magnetic resonance imaging brain morphometry studies in bipolar disorder. Biol. Psychiatry.

[bib40] Merz E.C., He X., Noble K.G. (2018). Anxiety, depression, impulsivity, and brain structure in children and adolescents. NeuroImage Clin..

[bib41] Michelini G., Barch D.M., Tian Y., Watson D., Klein D.N., Kotov R. (2019). Delineating and validating higher-order dimensions of psychopathology in the Adolescent Brain Cognitive Development (ABCD) study. Transl. Psychiatry.

[bib42] Moon C.-M., Kim G.-W., Jeong G.-W. (2014). Whole-brain gray matter volume abnormalities in patients with generalized anxiety disorder: voxel-based morphometry. Neuroreport.

[bib43] Muthén L.K., Muthén B.O. (1998-2017). Mplus User’s Guide.

[bib44] Narr K.L., Woods R.P., Lin J., Kim J., Phillips O.R., Del’Homme M., Caplan R., Toga A.W., McCracken J.T., Levitt J.G. (2009). Widespread cortical thinning is a robust anatomical marker for Attention Deficit/Hyperactivity Disorder (ADHD). J. Am. Acad. Child Adoles. Psychiatry.

[bib45] Nylund K.L., Asparouhov T., Muthén B.O. (2007). Deciding on the number of classes in latent class analysis and growth mixture modeling: a Monte Carlo simulation study. Struct. Eq. Model. Multidiscip. J..

[bib46] Olsen K.A., Rosenbaum B. (2006). Prospective investigations of the prodromal state of schizophrenia: review of studies. Acta Psychiatr. Scand..

[bib47] Peng W., Chen Z., Yin L., Jia Z., Gong Q. (2016). Essential brain structural alterations in major depressive disorder: a voxel-wise meta-analysis on first episode, medication-naive patients. J. Affect. Disord..

[bib48] Pessoa L. (2014). Understanding brain networks and brain organization. Phys. Life Rev..

[bib49] Powers A., Casey B.J. (2015). The adolescent brain and the emergence and peak of psychopathology. J. Infant Child Adoles. Psychother..

[bib50] Roberts H., Pozzi E., Vijayakumar N., Richmond S., Bray K., Deane C., Whittle S. (2021). Structural brain development and aggression: a longitudinal study in late childhood. Cogn. Affect. Behav. Neurosci..

[bib51] Rosso I.M., Cintron C.M., Steingard R.J., Renshaw P.F., Young A.D., Yurgelun-Todd D.A. (2005). Amygdala and hippocampus volumes in pediatric major depression. Biol. Psychiatry.

[bib52] Schienle A., Ebner F., Schäfer A. (2011). Localized gray matter volume abnormalities in generalized anxiety disorder. Eur. Arch. Psychiatry Clin. Neurosci..

[bib53] Schmaal L., Hibar D.P., Sämann P.G., Hall G.B., Baune B.T., Jahanshad N., Cheung J.W., van Erp T.G.M., Bos D., Ikram M.A., Vernooij M.W., Niessen W.J., Tiemeier H., Hofman A., Wittfeld K., Grabe H.J., Janowitz D., Bülow R., Selonke M., Veltman D.J. (2017). Cortical abnormalities in adults and adolescents with major depression based on brain scans from 20 cohorts worldwide in the ENIGMA Major Depressive Disorder Working Group. Mol. Psychiatry.

[bib54] Schmaal L., Veltman D.J., van Erp T.G.M., Sämann P.G., Frodl T., Jahanshad N., Loehrer E., Tiemeier H., Hofman A., Niessen W.J., Vernooij M.W., Ikram M.A., Wittfeld K., Grabe H.J., Block A., Hegenscheid K., Völzke H., Hoehn D., Czisch M., Hibar D.P. (2016). Subcortical brain alterations in major depressive disorder: findings from the ENIGMA Major Depressive Disorder working group. Mol. Psychiatry.

[bib55] Schwarz C.G., Gunter J.L., Wiste H.J., Przybelski S.A., Weigand S.D., Ward C.P., Senjem M.L., Vemuri P., Murray M.E., Dickson D.W., Parisi J.E., Kantarci K., Weiner M.W., Petersen R.C., Jack C.R. (2016). A large-scale comparison of cortical thickness and volume methods for measuring Alzheimer’s disease severity. NeuroImage Clin..

[bib56] Syal S., Hattingh C.J., Fouché J.-P., Spottiswoode B., Carey P.D., Lochner C., Stein D.J. (2012). Grey matter abnormalities in social anxiety disorder: a pilot study. Metab. Brain Dis..

[bib57] Talati A., Pantazatos S.P., Schneier F.R., Weissman M.M., Hirsch J. (2013). Gray matter abnormalities in social anxiety disorder: primary, replication, and specificity studies. Biol. Psychiatry.

[bib58] Tamnes C.K., Herting M.M., Goddings A.-L., Meuwese R., Blakemore S.-J., Dahl R.E., Güroğlu B., Raznahan A., Sowell E.R., Crone E.A., Mills K.L. (2017). Development of the cerebral cortex across adolescence: a multisample study of inter-related longitudinal changes in cortical volume, surface area, and thickness. J. Neurosci. Off. J. Soc. Neurosci..

[bib59] Valera E.M., Faraone S.V., Murray K.E., Seidman L.J. (2007). Meta-analysis of structural imaging findings in attention-deficit/hyperactivity disorder. Biol. Psychiatry.

[bib60] Varol E., Sotiras A., Davatzikos C. (2017). HYDRA: revealing Heterogeneity of imaging and genetic patterns through a multiple max-margin Discriminative Analysis framework. NeuroImage.

[bib61] Vijayakumar N., Allen N.B., Dennison M., Byrne M.L., Simmons J.G., Whittle S. (2017). Cortico-amygdalar maturational coupling is associated with depressive symptom trajectories during adolescence. NeuroImage.

[bib62] Waller R., Hawes S.W., Byrd A.L., Dick A.S., Sutherland M.T., Riedel M.C., Tobia M.J., Bottenhorn K.L., Laird A.R., Gonzalez R. (2020). Disruptive behavior problems, callous-unemotional traits, and regional gray matter volume in the Adolescent Brain and Cognitive Development Study. Biol. Psychiatry Cogn. Neurosci. Neuroimaging.

[bib63] Wang X., Cheng B., Luo Q., Qiu L., Wang S. (2018). Gray matter structural alterations in social anxiety disorder: a voxel-based meta-analysis. Front. Psychiatry.

[bib64] Wicherts J.M., Veldkamp C.L.S., Augusteijn H.E.M., Bakker M., van Aert R.C.M., van Assen M.A.L.M. (2016). Degrees of freedom in planning, running, analyzing, and reporting psychological studies: a checklist to avoid p-Hacking. Front. Psychology.

[bib65] Winkler A.M., Kochunov P., Blangero J., Almasy L., Zilles K., Fox P.T., Duggirala R., Glahn D.C. (2010). Cortical thickness or grey matter volume? The importance of selecting the phenotype for imaging genetics studies. NeuroImage.

[bib66] Zipursky A.R., Whittle S., Yücel M., Lorenzetti V., Wood S.J., Lubman D.I., Simmons J.G., Allen N.B. (2011). Pituitary volume prospectively predicts internalizing symptoms in adolescence. J. Child Psychol. Psychiatry Allied Discip..

