# Supplementary material for: Identifying profiles of brain structure and associations with current and future psychopathology in youth
Source: Dev Cogn Neurosci. 2021 Sep 14;51:101013. doi: 10.1016/j.dcn.2021.101013 (PMC8461345; doi:10.1016/j.dcn.2021.101013)
Supplement: Supplementary file 1 — Supplementary material [file mmc1.docx]

**Supplementary Materials Table of Contents**

| **Supplementary Table 1** | Grand CBCL Means | 2 |
| --- | --- | --- |
| **Supplementary Table 2** | Lifetime KSADS Prevalence | 2 |
| **Supplementary Figure 1** | Sample Identification Flow Chart | 2 |
| **Supplementary Profile Descriptions** |  | 3-5 |
| **Supplementary Figures 2-4** | Profile Membership Migration | 6-8 |
| **Supplementary Table 3** | Baseline/Follow-Up Correlations | 9 |
| **Supplementary Table 4** | Comparison with full sample | 10 |
| **Supplementary Table 5** | Full Sample LPA Fit | 10 |
| **Supplementary Table 6** | Profile Membership Consistency | 10 |
| **Supplementary Figure 5** | Full Sample 5-Profile Solution | 11 |
| **Supplementary Table 7** | OFC Volume Solution ROIs | 12 |
| **Supplementary Table 8** | OFC Volume Solution LPA Fit | 13 |
| **Supplementary Figures 6-15** | Solutions for OFC Volume LPA | 14-21 |
| **Supplementary Table 9** | CBCL Differences for OFC Volume Model | 22 |
| **Supplementary Table 10** | KSADS Differences for OFC Volume Model | 23 |

Supplementary Table 1

*Grand CBCL Means*

| **CBCL Dimension** | *Mean (SD)* |
| --- | --- |
| **Internalizing** | 2.49 (2.94) |
| **Externalizing** | 4.48 (5.54) |
| **Detachment** | 0.67 (1.17) |
| **Somatoform** | 1.12 (1.56) |
| **Neurodevelopmental** | 2.90 (3.56) |

Supplementary Table 2

*Lifetime KSADS Prevalence*

| **Diagnosis** | *Prevalence* |
| --- | --- |
| **Depression** | 6.47% |
| **Bipolar** | 7.01% |
| **Anxiety** | 37.24% |
| **OCD** | 10.91% |
| **Behavioral** | 15.36% |
| **ADHD** | 21.42% |

Supplementary Figure 1

*Sample Identification Flow Chart*

**Supplementary Profile Descriptions**

All profiles are described by how ROI thickness/volume differ in standard deviations from the grand mean.

In the 2-profile solution, Profile 1 (55%) had bilaterally small nucleus accumbens, caudate, and putamen regions (-.42 to -.50) as well as a small right amygdala (-.31) and right pallidum (-.48). Profile 2 (45%) had bilaterally large nucleus accumbens, caudate, putamen, and pallidum regions (.31-60), as well as a large right amygdala (.39), right hippocampus (.33), and right thalamus (.34). Thus, the 2-profile solution separated individuals into a small subcortical profile and a large subcortical profile.

In the 3-profile solution, Profile 1 (35%) was characterized by bilaterally small subcortical regions (-.34 to -.66), other than the left amygdala. Profile 2 (28%) was characterized by bilaterally small orbitofrontal regions (-.75 to -.86). Profile 3 (35%) was characterized by bilaterally large orbitofrontal regions (.36 to .49) and bilaterally large subcortical regions (.30 to .64), other than the left thalamus. Thus, the 3-profile solution separated individuals into a small subcortical profile, a small orbitofrontal profile, and a globally large profile.

In the 4-profile solution, Profile 1 (29%) was characterized by bilaterally small orbitofrontal regions (.66 to .74) and Profile 2 (30%) was characterized by bilaterally large orbitofrontal regions (.63 to .69). Profile 3 (22%) was characterized by bilaterally small subcortical regions (-.31 to -.93) and a small right lateral orbitofrontal cortex. Profile 4 (19%) was characterized by bilaterally large subcortical regions (.48 to .62), large caudate, nucleus accumbens, putamen, and right amygdala sizes (.43 to 1.01) and a large right medial (.37) and right lateral (.39) orbitofrontal cortex. Thus, the 4-profile solution contained profiles that differed primarily by groupings of large and small orbitofrontal and subcortical regions.

In the 5-profile solution, Profile 1 (20%) was characterized by bilaterally small subcortical regions (-.56 to -.92). Profile 2 (23%) was characterized by small orbitofrontal regions (-.72 to -.84). Profile 3 (19%) was characterized by bilaterally small amygdala and hippocampus (-.31 to -.41), bilaterally large caudate (.89, .90), and a large right pallidum (.57). Profile 4 (23%) was characterized by bilaterally large orbitofrontal regions (.61 to .73), bilaterally small caudate (-.33 to -.36), and small right pallidum (-.32). Profile 5 (15%) was characterized by bilaterally large subcortical regions (.47 to 1.08) and bilaterally large orbitofrontal regions (.34 to .44), other than the left medial orbitofrontal region (.27).

In the 6-profile solution, Profile 1 (12%) was characterized by small orbitofrontal regions (-.43 to -.59) and small subcortical regions (-.55 to -1.02). Profile 2 (18%) was characterized by large orbitofrontal regions (-.47 to -.61), other than the left medial orbitofrontal region (.29), and bilaterally small nucleus accumbens, caudate, putamen, and pallidum (-.45 to -.87), as well as a small right thalamus (-.30). Profile 3 (24%) was characterized by small orbitofrontal regions (-.72 to -.82). Profile 4 (22%) was characterized by bilaterally small amygdala and hippocampus (-.35 to -.39), and bilaterally large caudate (.55). Profile 5 (13%) was characterized by large orbitofrontal regions (.45 to .70), and bilaterally large nucleus accumbens, amygdala, hippocampus, and putamen (.60 to 1.20). Profile 6 (11%) was characterized by bilaterally large subcortical regions (.31 to 1.22), other than the L amygdala (.18). Thus, the 6-profile solution contained profiles that were characterized by orbitofrontal and subcortical groupings, variation between subcortical regions, and a profile with a large orbitofrontal regions and small subcortical regions.

In the 7-profile solution, Profile 1 (16%) was characterized by bilaterally small subcortical regions (-.56 to -.98) and a small R lateral orbitofrontal region (-.32). Profile 2 (13%) was characterized by bilaterally small orbitofrontal regions (-1.04 to -1.20), and a small right amygdala (-.32). Profile 3 (20%) was characterized by bilaterally small amygdala and hippocampus (-.32 to -.4), a bilaterally large caudate (.70, .72), a large left medial orbitofrontal cortex (.33), and a large right pallidum (.44). Profile 4 (19%) was characterized by a bilaterally large amygdala and hippocampus (.40 to .54) and a bilaterally small caudate (-.54, -.55). Profile 5 (12%) was characterized by bilaterally large orbitofrontal regions (.86 to .92), a bilaterally small nucleus accumbens, caudate, and pallidum (-.33 to -.50), as well as a small left thalamus (-.31) and a small right putamen (-.30). Profile 6 (11%) was characterized by bilaterally large orbitofrontal regions (.64 to .90), a bilaterally large nucleus accumbens, amygdala, caudate, hippocampus, and putamen (.42 to 1.20), as well as a large right pallidum (.47). Profile 7 (9%) was characterized by bilaterally large subcortical regions (.33 to 1.10), other than the left amygdala (.27), as well as a small left lateral orbitofrontal region (-.39). Thus, the 7-profile solution separated profiles by orbitofrontal groupings, subcortical groupings, and increased variability between subcortical regions.

In the 8-profile solution, Profile 1 (10%) was characterized by bilaterally small subcortical regions (-.37 to -1.11) and bilaterally small orbitofrontal regions (-.43 to -.57). Profile 2 (12%) was characterized by bilaterally small orbitofrontal regions (-.44 to -.49), a bilaterally large hippocampus and thalamus (.36 to .49), and a bilaterally small putamen (-.58, -.67). Profile 3 (12%) was characterized by a bilaterally small accumbens, caudate, putamen, pallidum, and thalamus (-.39 to -1.26). Profile 4 (16%) was characterized by bilaterally small orbitofrontal regions (-.65 to -.77), a bilaterally small caudate (-.43, -.45), and a bilaterally large putamen (.51, .55). Profile 5 (14%) was characterized by bilaterally small hippocampus (-.31, -.33), a small left amygdala (-.32), a bilaterally large caudate (1.15, 1.16), a large left accumbens (.42), and a large right putamen (.33) and pallidum (.66). Profile 6 (16%) was characterized by large orbitofrontal regions (.70 to .75). Profile 7 (12%) was characterized by bilaterally large orbitofrontal regions (.47 to .70), and a bilaterally large nucleus accumbens, amygdala, hippocampus, and putamen (.45 to 1.13). Profile 8 (7%) was characterized by bilaterally large subcortical regions (.51 to 1.46). Thus, the 8-profile solution separated profiles by orbitofrontal groupings and a combination of subcortical groupings and subcortical region variation.

In the 9-profile solution, Profile 1 (7%) was characterized by bilaterally small subcortical regions (-.40 to -1.26) and bilaterally small orbitofrontal regions (-.63 to -.89). Profile 2 (11%) was characterized by bilaterally small orbitofrontal regions (-.37to -.44), a bilaterally large hippocampus and thalamus (.46 to .53), and a bilaterally small putamen (-.61, -.69). Profile 3 (10%) was characterized by bilaterally large orbitofrontal regions (.62 to .73) and bilaterally large subcortical regions (.43 to .89). Profile 4 (16%) was characterized by bilaterally large orbitofrontal regions (.49 to .55) and a bilaterally small accumbens, caudate, putamen, pallidum, and thalamus (-.41 to -.83). Profile 5 (16%) was characterized by bilaterally large orbitofrontal regions (.50 to .75), a bilaterally large caudate (.68, .69), and a large right pallidum (.32). Profile 6 (15%) was characterized by bilaterally small orbitofrontal regions (-.55 to -.68), a bilaterally small caudate (-.50, -.53), and a bilaterally large putamen (.57, .60). Profile 7 (7%) was characterized by small orbitofrontal regions (-.52 to -.59) and a bilaterally large accumbens, caudate, putamen, and pallidum (.32 to 1.30). Profile 8 (10%) was characterized by bilaterally large orbitofrontal regions (.49 to .75), and a bilaterally large nucleus accumbens, amygdala, hippocampus, and putamen (.47 to 1.21). Profile 9 (7%) was characterized by bilaterally large subcortical regions (.51 to 1.42) and large right orbitofrontal regions (.42, .42). Thus, the 9-profile solution separated profiles by orbitofrontal groupings and a combination of subcortical groupings and subcortical region variation.

For Supplementary Figures, arrow thickness & darkness corresponds to percentage of individuals in prior solution profile that moved to current solution profile.

Supplementary Figure 2

*Profile Membership Migration Between Solutions 1-4*


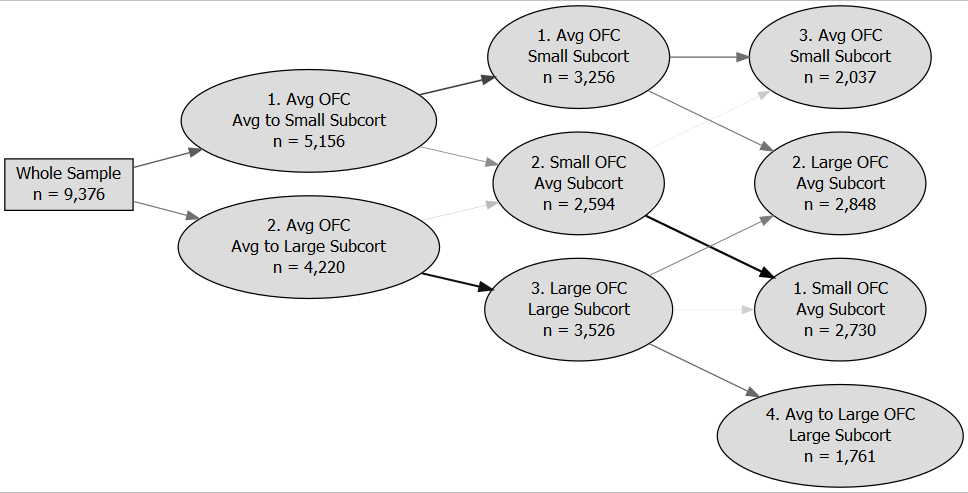


Supplementary Figure 3

*Profile Membership Migration Between Solutions 4-7*


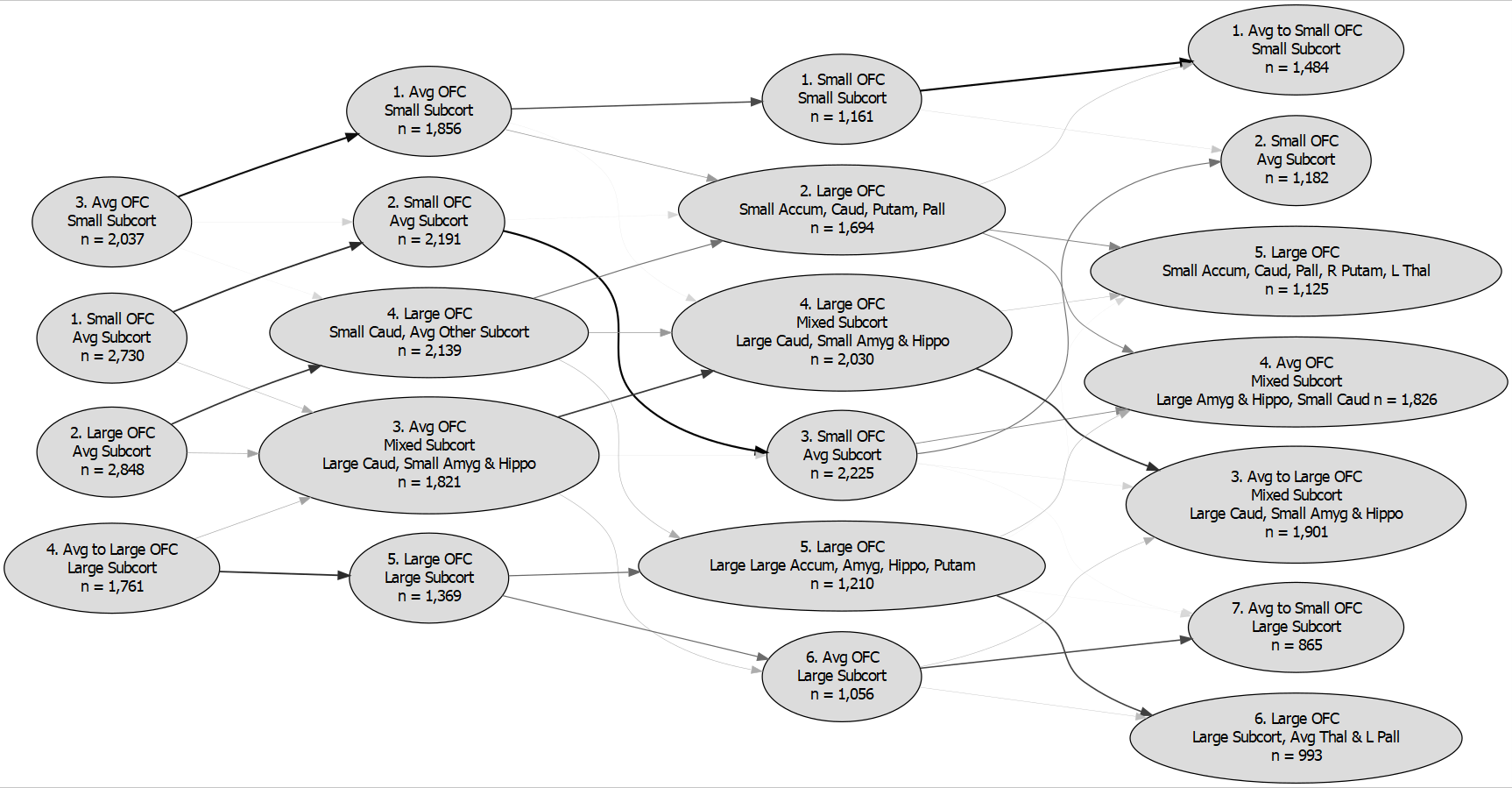


Supplementary Figure 4

*Profile Membership Migration Between Solutions 7-9*


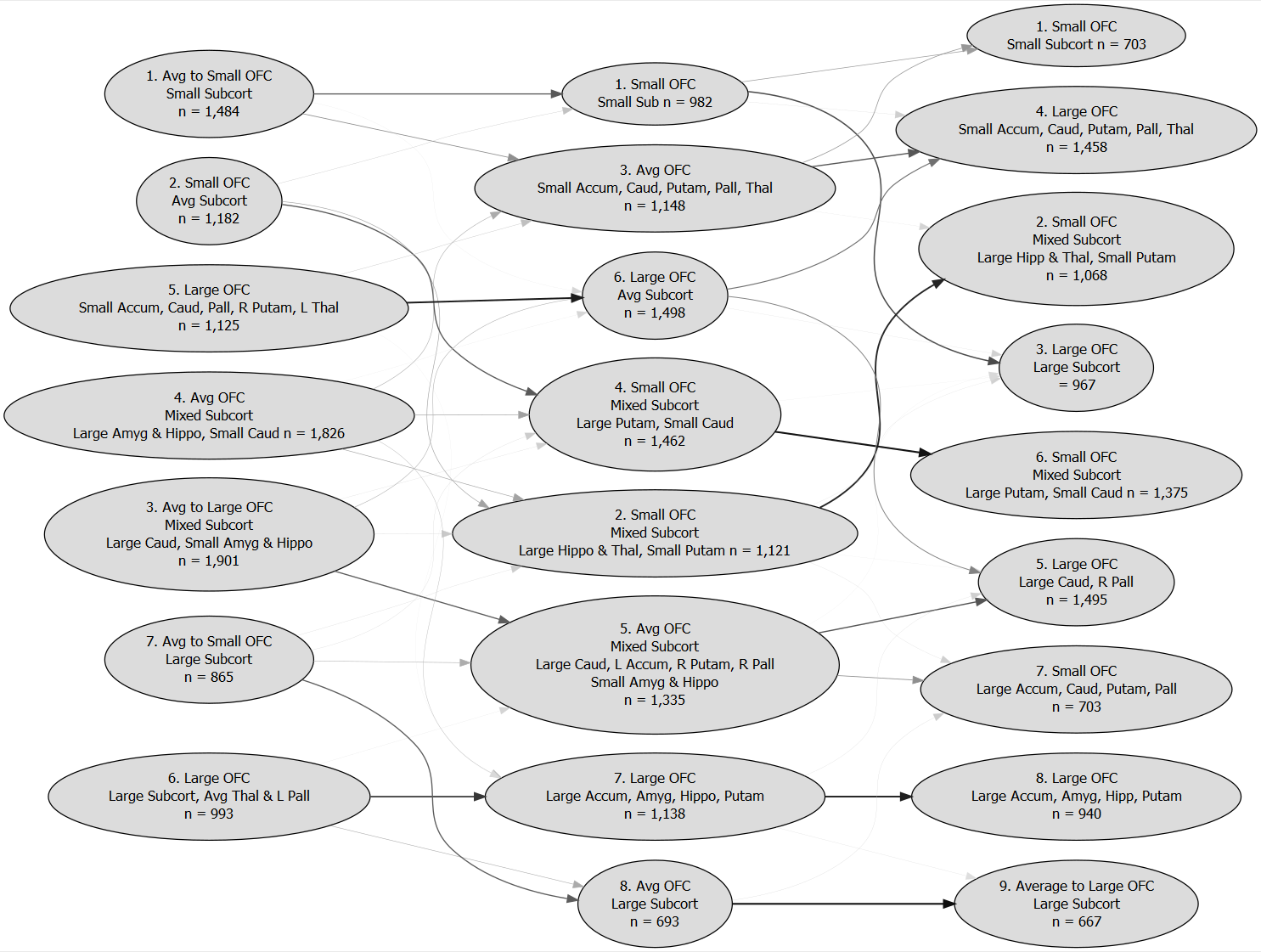


Supplementary Table 3

*Correlations coefficients of baseline and follow-up measures within profiles*

| Profile | Intern. | Extern. | Detach. | Somat. | Neuro. | PDQ Sum | PDQ Sev. |
| --- | --- | --- | --- | --- | --- | --- | --- |
| 1 | .68 | .75 | .59 | .53 | .77 | .42 | .42 |
| 2 | .68 | .77 | .62 | .54 | .74 | .50 | .50 |
| 3 | .67 | .73 | .61 | .51 | .77 | .46 | .46 |
| 4 | .67 | .73 | .58 | .53 | .72 | .47 | .47 |
| 5 | .67 | .74 | .57 | .56 | .73 | .50 | .50 |

*Note.* Profile 1: Reduced Subcortical Volume. Profile 2: Reduced OFC Thickness. Profile 3: Reduced Limbic/Elevated Striatal Volume. Profile 4: Elevated OFC Thickess/Reduced Striatal Volume. Profile 5: Elevated OFC Thickness & Subcortical Volume.

*Abbreviations.* Intern. = Internalizing. Detach. = Detachment. Somat. = Somatoform. Neuro. = Neurodevelopmental. PDQ Sev. = PDQ Severity

**Comparison to LPA with sample including siblings (*N* = 11,076)**

Supplementary Table 4

*Sample Comparison with Full ABCD sample, after excluding failed FreeSurfer quality control*

|  | **Sample with 1 Participant per Family (*N* = 9,376)**  ***M (SD)*** | **Full sample (*N* = 11,076)**  ***M (SD)*** |
| --- | --- | --- |
| **Sex** | 47.5% Female | 47.9% Female |
| **Age** | 9.91 (0.62) | 9.92 (0.62) |
| **Internalizing** | 2.49 (2.94) | 2.41 (2.89) |
| **Externalizing** | 4.49 (5.55) | 4.40 (5.49) |
| **Detachment** | 0.67 (1.17) | 0.66 (1.17) |
| **Somatoform** | 1.12 (1.56) | 1.09 (1.54) |
| **Neurodevelopmental** | 2.91 (3.56) | 2.81 (3.50) |

Supplementary Table 5

*Full Sample LPA Fit Criteria*

| **Solution** | **AIC** | **BIC** | **aBIC** | **AICC** | **Parameters** | **Entropy** | **LMR-LRT *p*** |
| --- | --- | --- | --- | --- | --- | --- | --- |
| 2 | 547528.4 | 547930.6 | 547755.8 | 547529.0 | 55 | 0.774 | **< .001** |
| 3 | 542029.5 | 542570.5 | 542335.4 | 542030.5 | 74 | 0.749 | 0.437 |
| 4 | 537162.7 | 537842.7 | 537547.1 | 537164.2 | 93 | 0.753 | 0.080 |
| 5* | 533686.9 | 534505.8 | 534149.9 | 533689.2 | 112 | 0.755 | **.0003** |
| 6 | 531655.3 | 532613.1 | 532196.8 | 531658.4 | 131 | 0.758 | 0.241 |
| 7 | 529838.9 | 530935.7 | 530459.0 | 529843.1 | 150 | 0.754 | 0.309 |
| 8 | 528171.8 | 529407.5 | 528870.4 | 528177.1 | 169 | 0.771 | 0.596 |
| 9 | 526706.5 | 528081.2 | 527483.7 | 526713.1 | 188 | 0.767 | 0.493 |

*The 5-Profile solution was selected in the sample utilized in the main study that only included 1 participant per family. In this full sample, the 5-Profile solution also shows improved fit relative to the 4-Profile solution.

*Abbreviations*: Akaike Information Criteria (AIC), Bayesian Information Criteria (BIC), sample-size adjusted BIC (aBIC), corrected AIC (AICC), Lo-Mendell-Rubin Likelihood Ratio Test (LMR-LRT)

Supplementary Table 6

*Consistency of Most Likely Profile Membership Across Samples*

| Profile | Consistency (%) |
| --- | --- |
| 1 | 96.5% |
| 2 | 95.2% |
| 3 | 98.4% |
| 4 | 95.6% |
| 5 | 99.2% |

*Note.* Consistency represents the percentage of individuals in initial sample with the same most likely profile membership in the larger sample that included siblings.

Supplementary Figure 5

*Full Sample 5-Profile Solution*


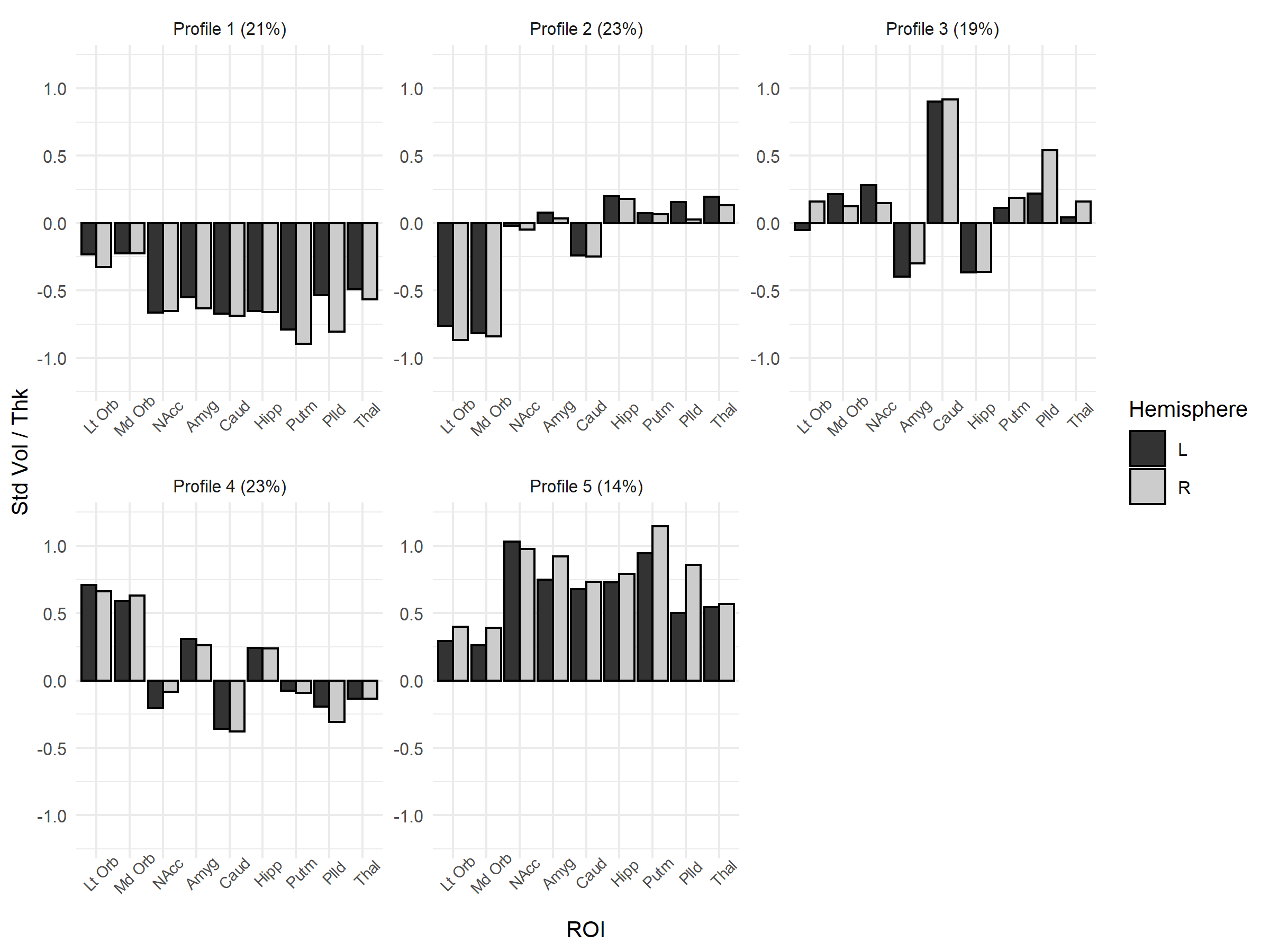


**LPA results using OFC Volume instead of OFC thickness, as initially pre-registered**

We present this data as the initial pre-registration included OFC volume, which we later changed to OFC thickness in consistency with field standards for cortical brain structure imaging.

Supplementary Table 7

*Order of ROIs in OFC volume solution plots*

| 0 | L Lateral OFC |
| --- | --- |
| 1 | L Medial OFC |
| 2 | L Accumbens |
| 3 | L Amygdala |
| 4 | L Caudate |
| 5 | L Hippocampus |
| 6 | L Putamen |
| 7 | L Pallidum |
| 8 | R Lateral OFC |
| 9 | R Medial OFC |
| 10 | R Accumbens |
| 11 | R Amygdala |
| 12 | R Caudate |
| 13 | R Hippocampus |
| 14 | R Pallidum |
| 15 | R Thalamus |
| 16 | R Putamen |
| 17 | L Thalamus |

Supplementary Table 8

*OFC Volume Models Fit Criteria*

| **# Profiles** | **LL** | **AIC** | **BIC** | **aBIC** | **AICC** | **Parameters** | **Entropy** | **LMR-LRT *p*** |
| --- | --- | --- | --- | --- | --- | --- | --- | --- |
| **2** | -234105 | 468319.5 | 468712.5 | 468537.8 | 468320.2 | 55 | 0.708 | 0.4313 |
| **3** | -231620 | 463388 | 463916.8 | 463681.6 | 463389.2 | 74 | 0.728 | 0.4942 |
| **4** | -230144 | 460474.2 | 461138.8 | 460843.3 | 460476.1 | 93 | 0.76 | 0.503 |
| **5** | -229068 | 458360.3 | 459160.7 | 458804.8 | 458363.1 | 112 | 0.752 | 0.5198 |
| **6** | -227953 | 456168.1 | 457104.2 | 456687.9 | 456171.8 | 131 | 0.763 | 0.4682 |
| **7** | -227209 | 454718.7 | 455790.6 | 455313.9 | 454723.6 | 150 | 0.763 | 0.5346 |
| **8** | -226534 | 453405.3 | 454613 | 454075.9 | 453411.5 | 169 | 0.767 | 0.5125 |
| **9** | -225913 | 452202.5 | 453546 | 452948.5 | 452210.3 | 188 | 0.775 | 0.5253 |

**Supplementary Figures 6-15: Solutions with OFC Volume**

*2 Profile Solution*


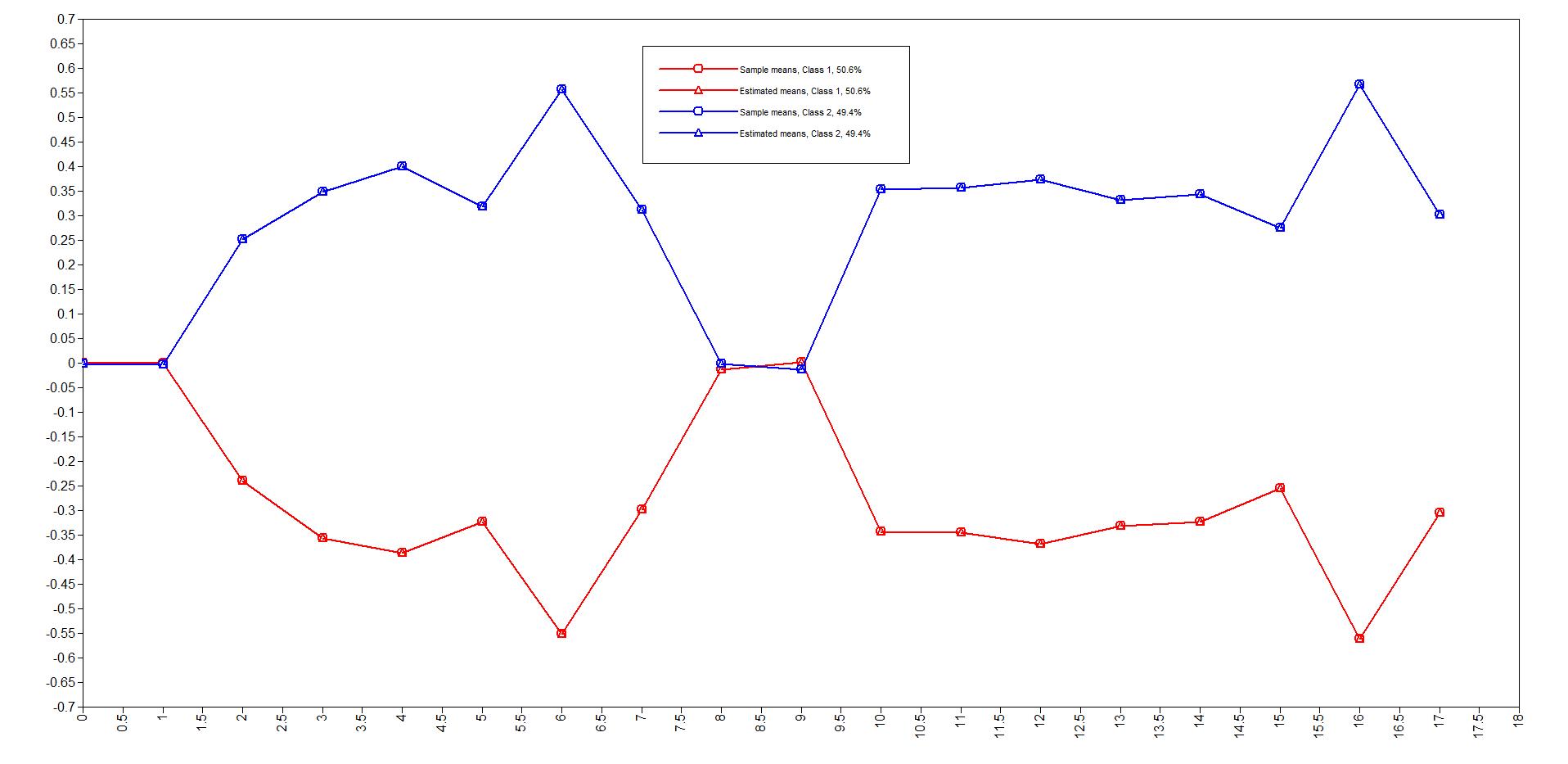


*3 Profile Solution*


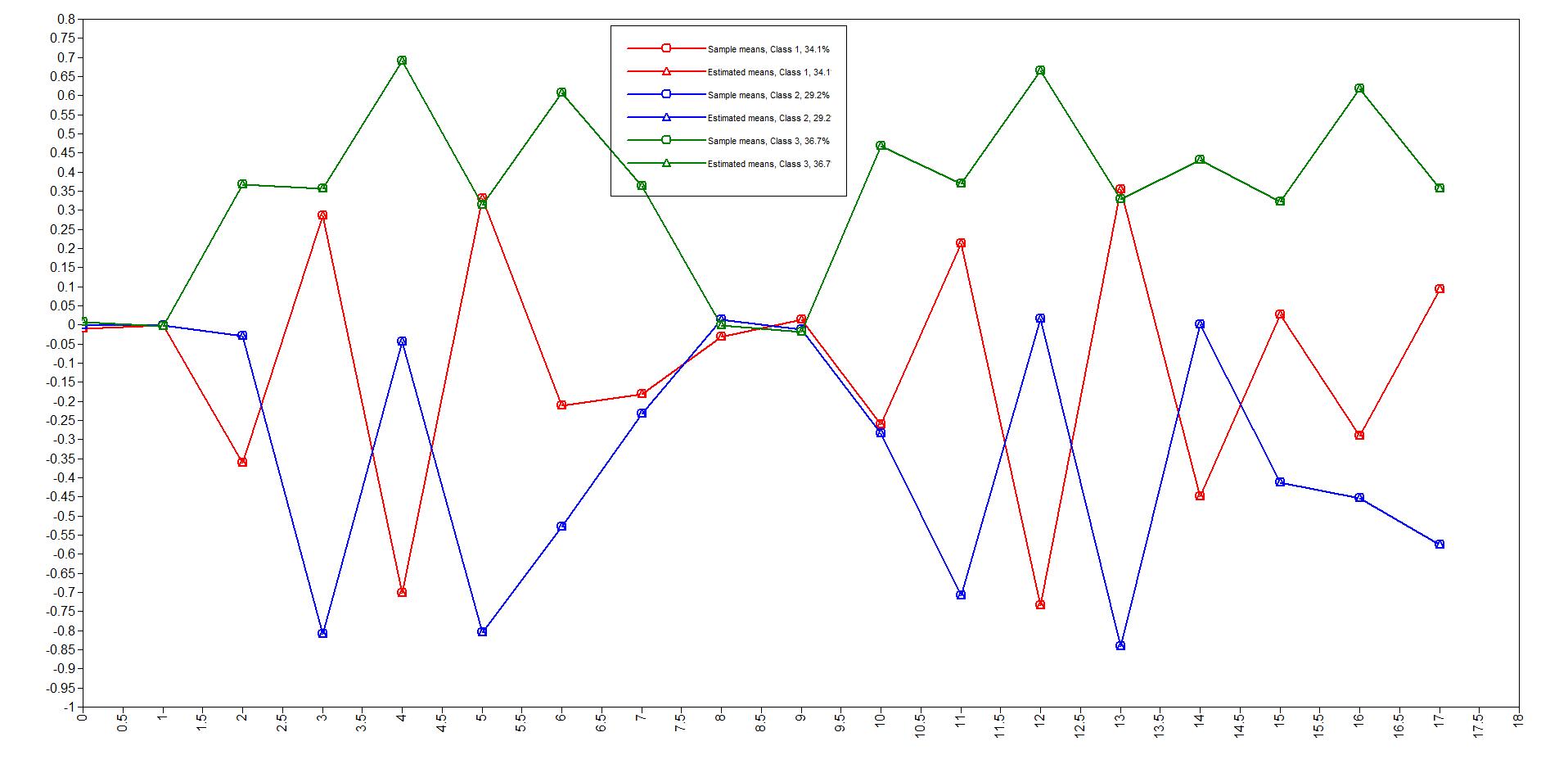


*4 Profile Solution*


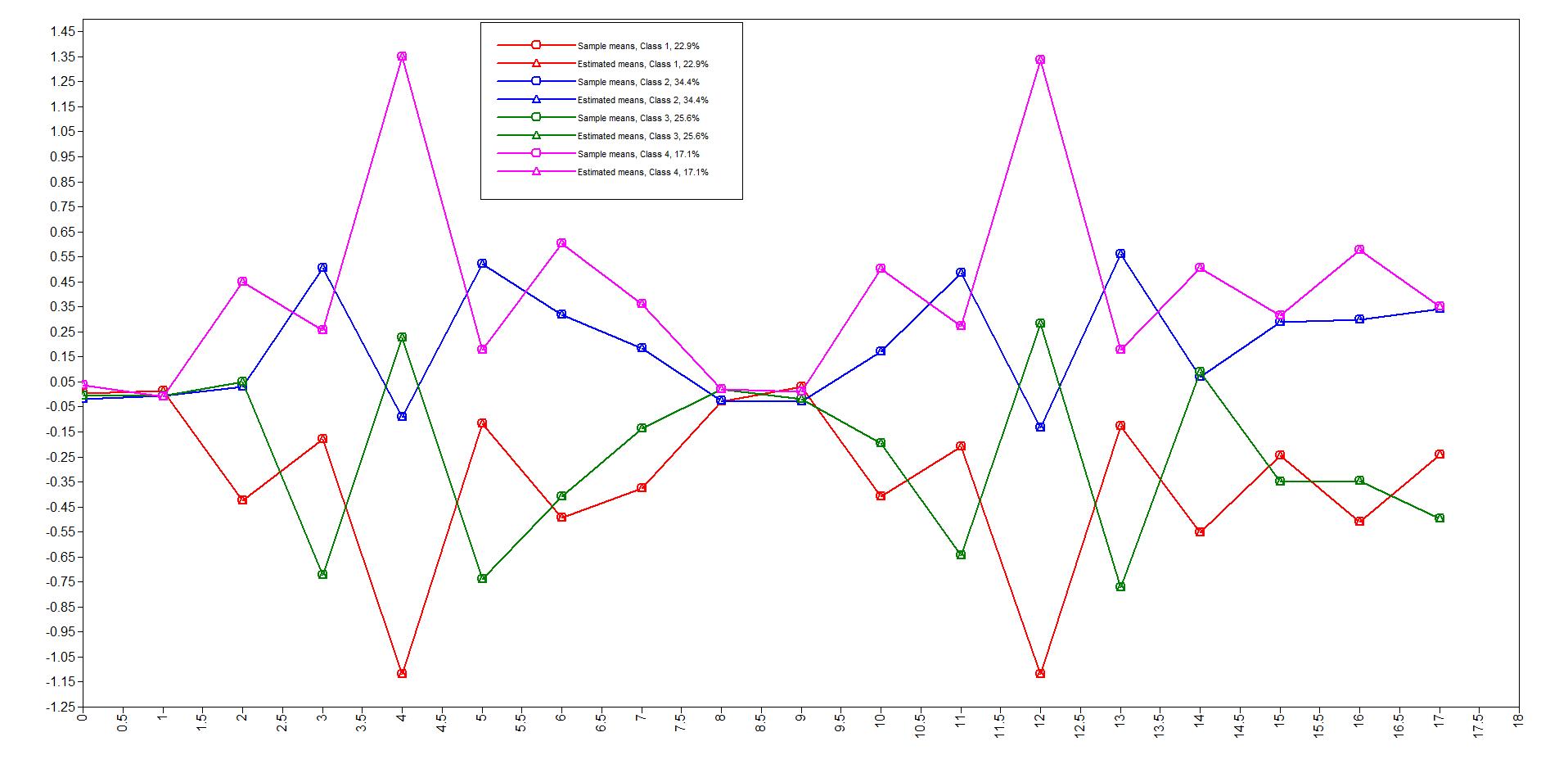


*5 Profile Solution*


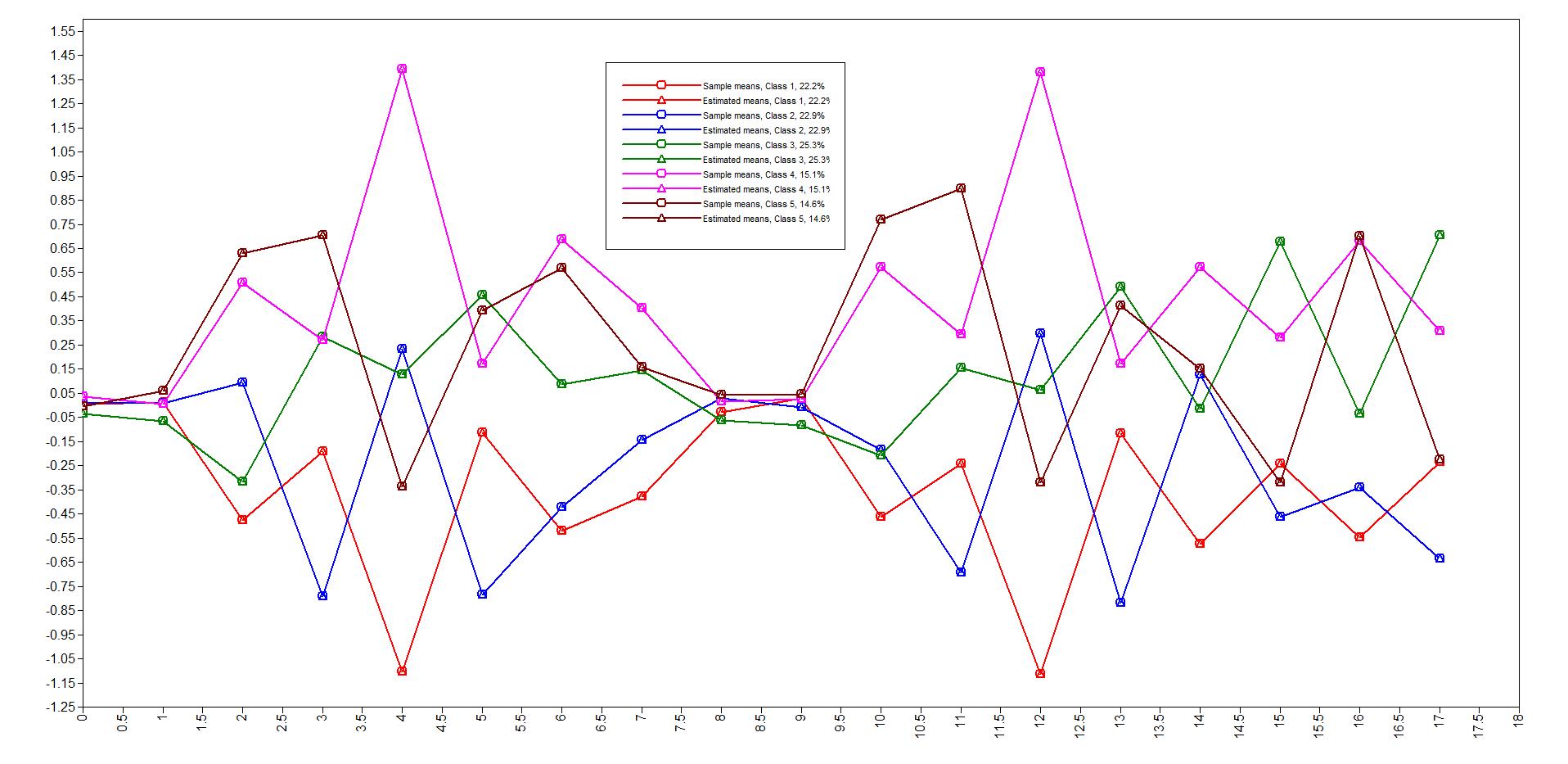


*6 Profile Solution*


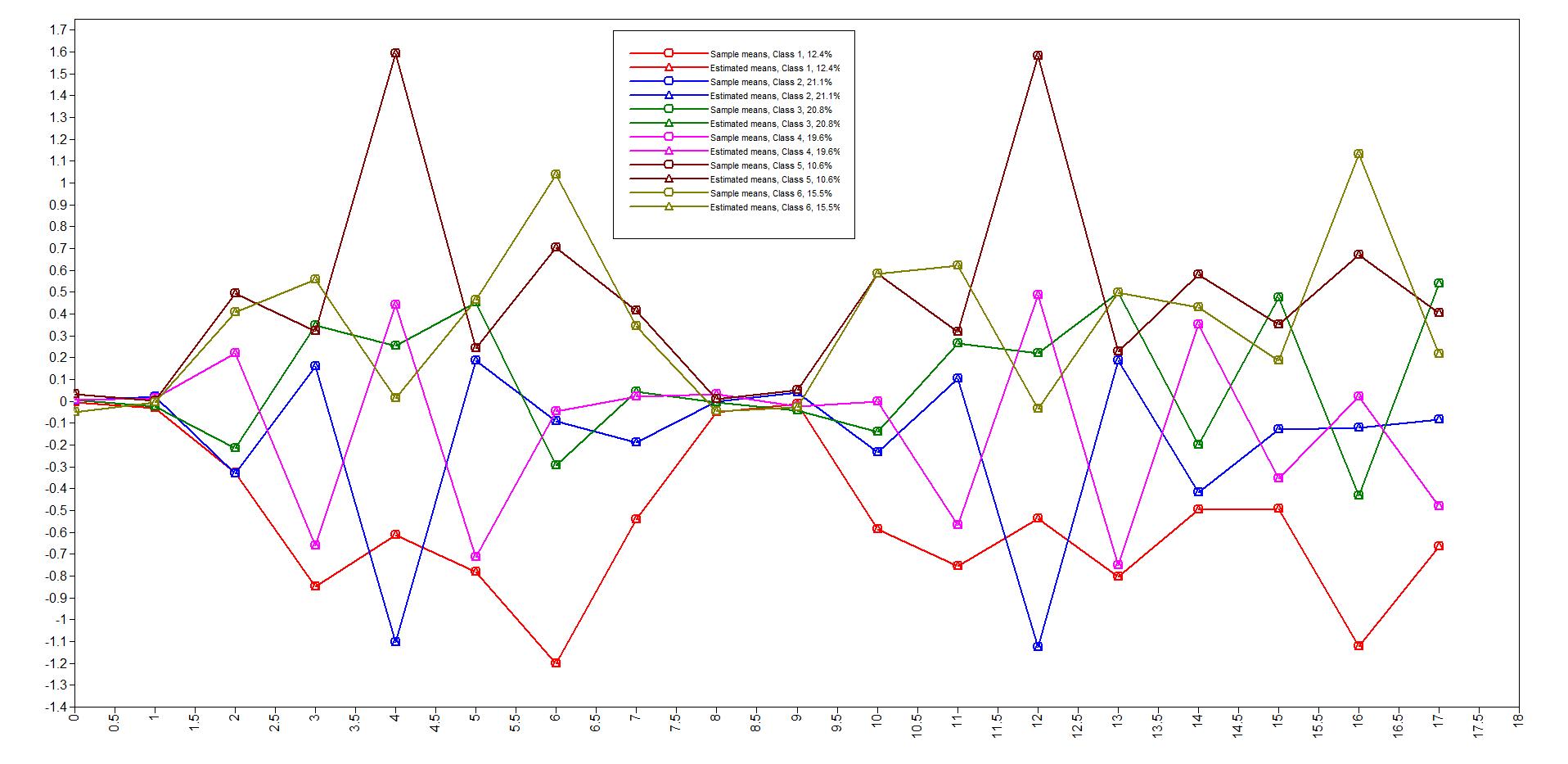


*7 Profile Solution*


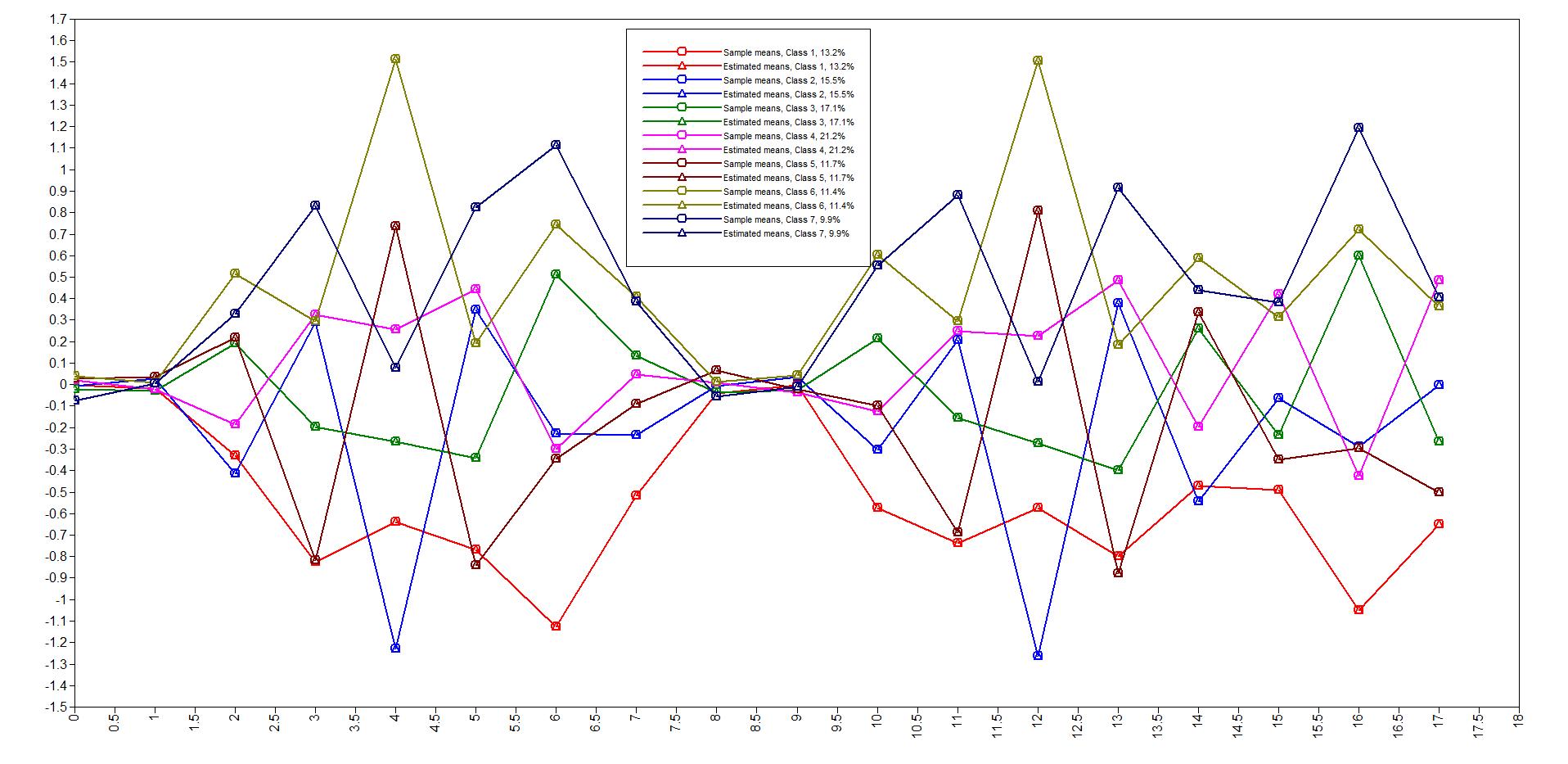


*8 Profile Solution*


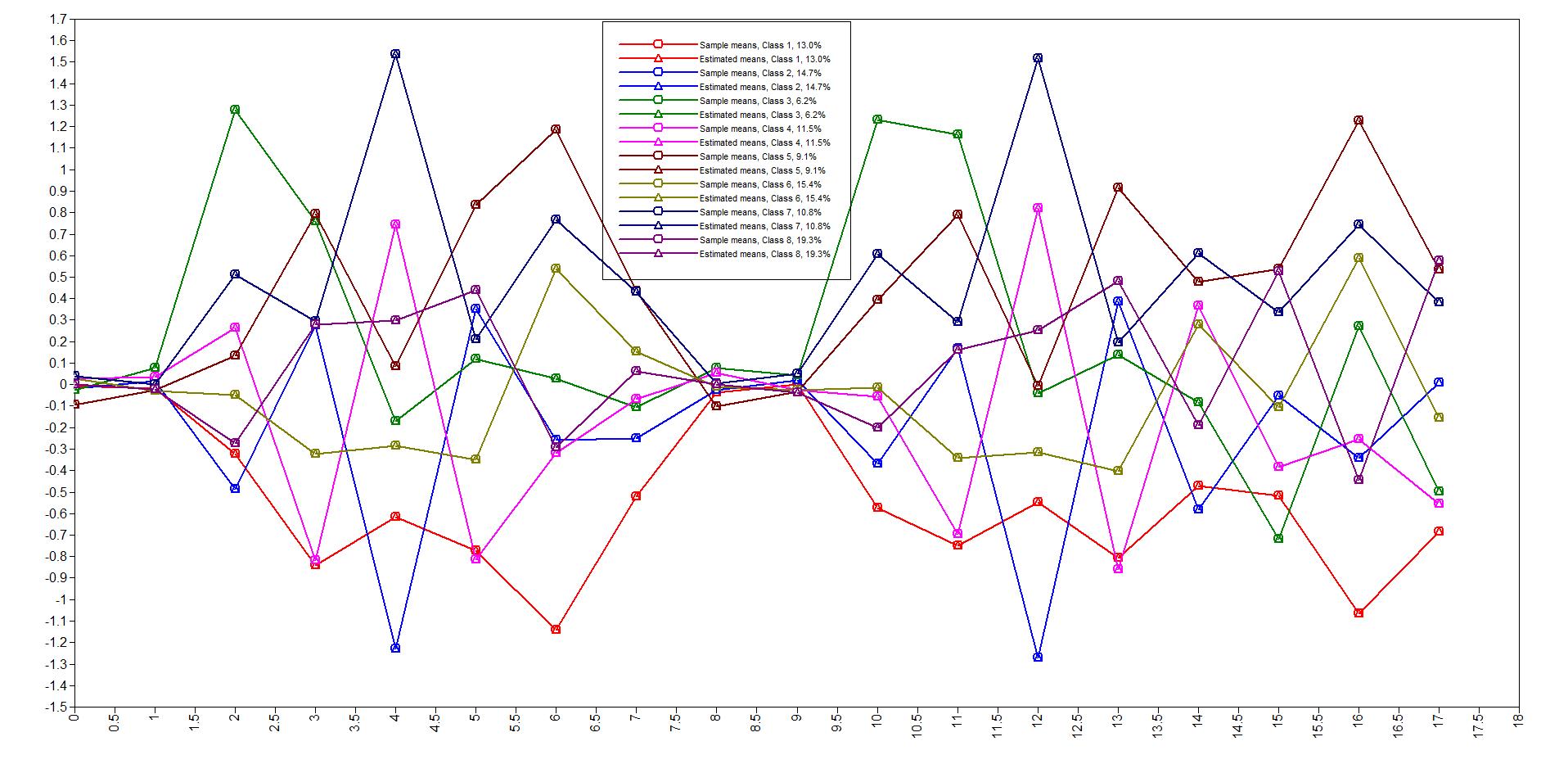


*9 Profile Solution*


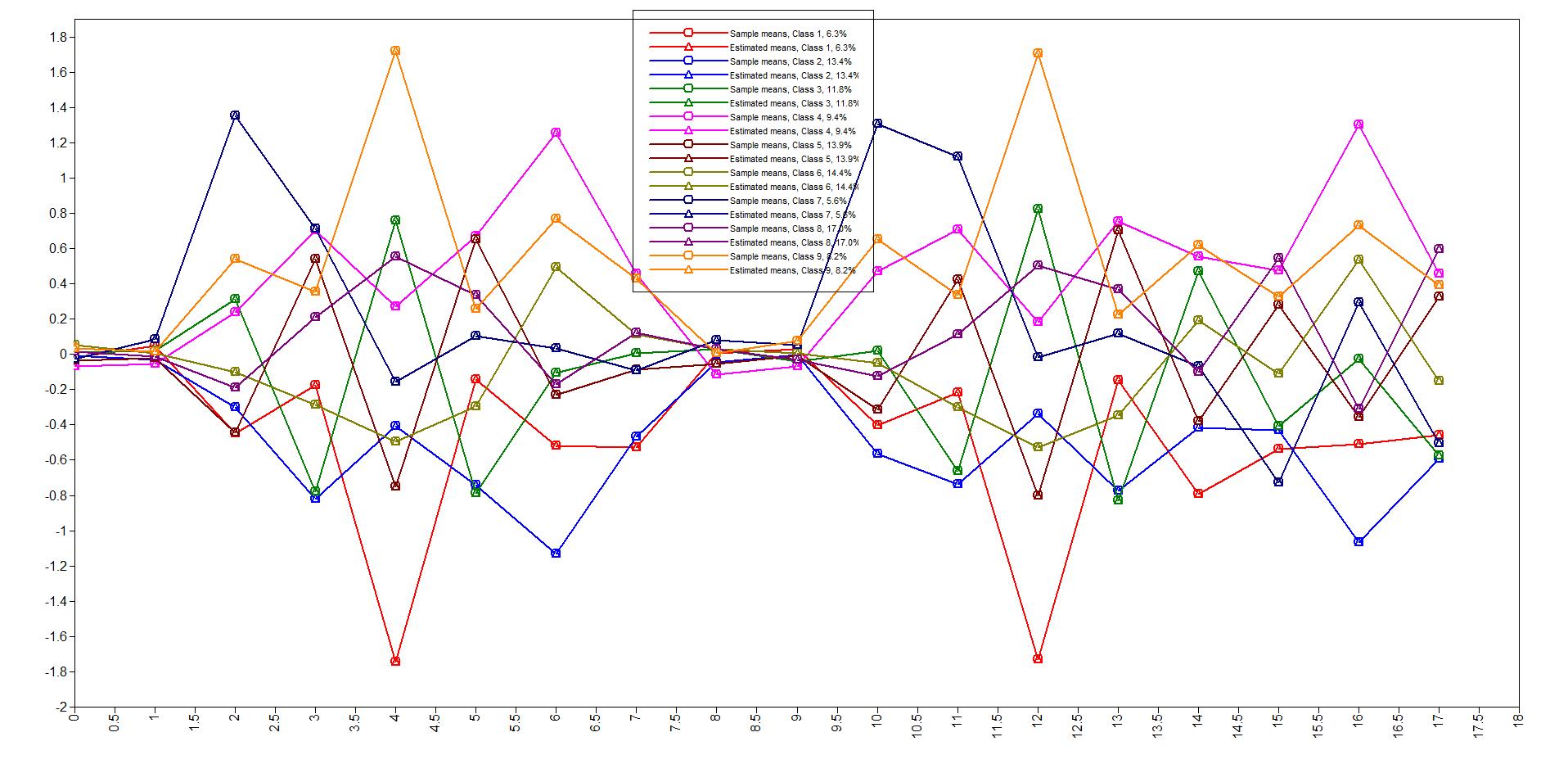


To examine what the results of psychopathology differences between profiles would have been if we had followed the preregistered volume analysis plan, we run supplemental post hoc analyses, selecting the 5-profile solution for comparison to the results presented in the main text. This solution also showed significant differences across CBCL and KSADS measures, though the results differ from that of the model in the main text. As there were some important differences in the classes identified between the profiles reported in the main text and those that were originally preregistered (i.e., there was a more prominent influence of OFC regions distinguishing classes in the analyses in the main text), these changes are expected.

Supplementary Table 9. CBCL Differences for OFC Volume Model: 5-Profile Solution

| Measure | Profile 1  *M (SD)* | Profile 2  *M (SD)* | Profile 3  *M (SD)* | Profile 4  *M (SD)* | Profile 5  *M (SD)* | Wald χ^2^ |
| --- | --- | --- | --- | --- | --- | --- |
| Internalizing | 2.44 (3)_a,b_ | 2.81 (3.15)_c_ | 2.32 (2.88)_b_ | 2.7 (2.97)_a,c_ | 2.68 (2.89)_a,b,c_ | 18.44** |
| Externalizing | 4.69 (5.97) | 4.96 (5.65) | 4.48 (5.81) | 4.53 (5.59) | 4.68 (5) | 5.55 |
| Detachment | 0.74 (1.27)_a,b,c_ | 0.78 (1.26)_c_ | 0.61 (1.15)_a,b_ | 0.63 (1.08)_b_ | 0.82 (1.29)_a,c_ | 35.93*** |
| Somatoform | 1.14 (1.63) | 1.19 (1.63) | 1.08 (1.56) | 1.3 (1.72) | 1.22 (1.54) | 8.89 |
| Neurodevelop. | 3.24 (3.98)_b,c_ | 3.24 (3.58)_b_ | 2.86 (3.57)_a_ | 2.89 (3.49)_a,c_ | 2.94 (3.51)_a,c_ | 23.85*** |

*Note.* * p < .05. ** p < .01. *** p < .001. For each row with a significant omnibus test, significant post-hoc differences are reflected by differing subscripts. Neurodevelop. = Neurodevelopmental. Degrees of Freedom: df = 4, 9363 for all tests.

Supplementary Table 10. KSADS Differences for OFC Volume Model: 5-Profile Solution

| Diagnosis | Profile 1  *Prob (CI)* | Profile 2  *Prob (CI)* | Profile 3  *Prob (CI)* | Profile 4  *Prob (CI)* | Profile 5  *Prob (CI)* | Wald χ^2^ |
| --- | --- | --- | --- | --- | --- | --- |
| Depression | 0.07 (0.05-0.1)_a,b_ | 0.09 (0.08-0.1)_b_ | 0.05 (0.04-0.07)_a_ | 0.07 (0.05-0.09)_a,b_ | 0.05 (0.04-0.08)_a_ | 24.24** |
| Bipolar | 0.08 (0.06-0.11) | 0.07 (0.06-0.09) | 0.06 (0.05-0.08) | 0.08 (0.05-0.11) | 0.09 (0.07-0.11) | 5.83 |
| Anxiety | 0.38 (0.34-0.43) | 0.39 (0.36-0.42) | 0.37 (0.34-0.41) | 0.4 (0.36-0.44) | 0.37 (0.33-0.42) | 3.20 |
| OCD | 0.1 (0.08-0.13)_a,c_ | 0.11 (0.1-0.13)_a,c_ | 0.1 (0.08-0.12)_c_ | 0.16 (0.12-0.2)_b_ | 0.14 (0.11-0.17)_a,b_ | 13.21* |
| Behavioral | 0.16 (0.13-0.2) | 0.15 (0.12-0.19) | 0.16 (0.14-0.19) | 0.15 (0.12-0.2) | 0.13 (0.11-0.17) | 2.87 |
| ADHD | 0.23 (0.2-0.26) | 0.22 (0.19-0.25) | 0.22 (0.19-0.24) | 0.19 (0.16-0.23) | 0.19 (0.16-0.23) | 3.41 |

*Note.* * p < .05. ** p < .01. *** p < .001. For each row, significant post-hoc differences are reflected by differing subscripts. Degrees of Freedom: df = 4, 9363 for all tests.
